# The impact of chromium toxicity on the yield and quality of rice grains produced under ambient and elevated levels of CO_2_

**DOI:** 10.3389/fpls.2023.1019859

**Published:** 2023-03-07

**Authors:** Hamada AbdElgawad, Afrah E. Mohammed, Jesper R. van Dijk, Gerrit T. S. Beemster, Modhi O. Alotaibi, Ahmed M. Saleh

**Affiliations:** ^1^ Integrated Molecular Plant Physiology Research, Department of Biology, University of Antwerp, Antwerp, Belgium; ^2^ Botany and Microbiology Department, Faculty of Science, Beni-Suef University, Beni-Suef, Egypt; ^3^ Department of Biology, College of Science, Princess Nourah bint Abdulrahman University, Riyadh, Saudi Arabia; ^4^ Ecosystem Management, Department of Biology, University of Antwerp, Antwerp, Wilrijk, Belgium; ^5^ Department of Botany and Microbiology, Faculty of Science, Cairo University, Giza, Egypt

**Keywords:** chromium, rice, yield, pollution, stress, eCO2

## Abstract

Rice is a highly valuable crop consumed all over the world. Soil pollution, more specifically chromium (Cr), decreases rice yield and quality. Future climate CO_2_ (eCO_2_) is known to affect the growth and yield of crops as well as the quality parameters associated with human health. However, the detailed physiological and biochemical responses induced by Cr in rice grains produced under eCO_2_ have not been deeply studied. Cr (200 and 400 mg Cr^6+^/Kg soil) inhibited rice yield and photosynthesis in Sakha 106, but to less extend in Giza 181 rice cultivar. Elevated CO_2_ reduced Cr accumulation and, consequently, recovered the negative impact of the higher Cr dose, mainly in Sakha 106. This could be explained by improved photosynthesis which was consistent with increased carbohydrate level and metabolism (starch synthases and amylase). Moreover, these increases provided a route for the biosynthesis of organic, amino and fatty acids. At grain quality level, eCO_2_ differentially mitigated Cr stress-induced reductions in minerals (e.g., P, Mg and Ca), proteins (prolamin, globulin, albumin, glutelin), unsaturated fatty acids (e.g., C20:2 and C24:1) and antioxidants (phenolics and total antioxidant capacity) in both cultivars. This study provided insights into the physiological and biochemical bases of eCO_2_-induced grain yield and quality of Cr-stressed rice.

## 1 Introduction

Contamination with heavy metals (HMs) is a critical global environmental problem since their concentrations exceeded the allowable thresholds affecting the quality of soil and crops leading to human health problems (Rai et al., 2019). The naturally occurring heavy metal, chromium (Cr), is beneficial for some plants and animals in trace amounts, however, at higher concentrations it could be a hazardous environmental contaminant (Srivastava et al., 2021). According to Environmental Protection Agency and the International Agency for Research on Cancer, Cr has been classified among the top harmful environmental pollutants and human carcinogens (Tchounwou et al., 2012). The metallic forms, chromite (Cr III) and chromate (Cr VI) are the most stable forms of Cr available in the environment (Vitale et al., 1997). The valence state of Cr affects its toxicity, Cr VI is considered as the more soluble and mobile form at all pH conditions which enhances its bioavailability leading to higher toxicity in relation to Cr III (Oliveira, 2012). Cr III can be oxidized to Cr VI form in the cell which disturbs cell components and integrity (Sharma et al., 2020).

Natural sources and several anthropogenetic activities have resulted in high Cr release into water, soil, and air (Wakeel et al., 2020). Cr contaminated soils lead to phytotoxicity and substantial reduction in the growth and yield quality characteristics (Shahid et al., 2017). Morphological, biochemical, physiological, and molecular changes were reported in *Arabidopsis thaliana* as affected by Cr (Castro et al., 2007; Martínez-Trujillo et al., 2014; Eleftheriou et al., 2015). Plant root absorbs and accumulates Cr from the soil and induces its availability in the aerial plant parts, *via* an inactive pathway, consequently, this could affect human health *via* the food chain (Giri and Singh, 2017). Cr absorption was found to reduce the ability of plant roots for essential nutrients uptake (Wakeel et al., 2020). Once inside the plant, Cr induces phytotoxicity both by immediate Cr-plant interaction, leading to metabolic pathways alterations, and *via* generation and accumulation of reactive oxygen species (ROS), thus, oxidative damage (Arif et al., 2019; Wakeel et al., 2020).

Rice (*Oryza sativa* L.) is a highly valuable food crop for the world’s population. Rice is rich in antioxidants, proteins, carbohydrates, micronutrients, and certain fatty acids (Panhwar et al., 2015). It feeds about 50% of the world population, but is highly affected by stress factors such as HMs (Arif et al., 2019). An earlier study by Zhu et al. (2008) indicated minimal changes in grain milling quality and morphology in Cr and zinc contaminated soils. However, Basit et al. (2021) reported adverse effects in plant biomass and photosynthetic rate besides enhancement of ROS levels and antioxidant enzymes under Cr toxicity for two rice varieties. Cr toxicity reduced rice biomass, photosynthetic pigments, and seedling growth besides ATP content and related enzymes (Ma et al., 2016). Such adverse effects in rice quality and yield, when cultivated in Cr polluted soil, urge the needs to develop growth conditions aimed at mitigating this negative impact on plants for sustainable rice production.

Elevated CO_2_ (eCO_2_) level has a direct effect on crop growth and yield as well as crop quality parameters associated with human health and food safety (Luo et al., 2019). The greatest outcome of eCO_2_ on plants is photosynthetic rate increment, thus improving the carbon fixation that may provide the carbohydrate beyond the plant’s need leading to increased biomass and yield quality (Senthil-Nathan, 2021). Plant photosynthesis, biomass, growth, and grain yield were enhanced in rice as positive consequences to eCO_2_-induced carbon accumulation (Senthil-Nathan, 2021). Consequently, the equilibrium in carbon nutrients may regulate the distribution of plant secondary metabolites (Wang et al., 2019). Becker and Kläring (2016) indicated that eCO_2_ could enhance red leaf lettuce quality *via* improvements in the accumulation of antioxidant compounds due to increment in their precursors (soluble sugars). Exudation of dissolved organic carbon into the soil, by plant roots, is increased due to eCO_2_ (Rosado-Porto et al., 2021). Interestingly, previous studies confirmed the mitigating effect of eCO_2_ against plant oxidative stress under different environmental limitations (Pérez-López et al., 2009; Abdelgawad et al., 2015; Shabbaj et al., 2022). In this context, in soil contaminated with Cu and Cd, eCO_2_ had affected HMs distribution in plants and soil and consequently, affected the production safety and quality for rice and wheat grown there (Guo et al., 2011). Kim and Kang (2011) reported that eCO_2_ increased the pine seedlings’ biomass and ability in lead uptake, besides high changes in tissue metabolites were noted. eCO_2_ boosted the HMs detoxification system in C3 and C4 species under indium oxide nanoparticles (Shabbaj et al., 2022). The impact of eCO_2_ on some vegetables’ nutritional quality is well documented in a meta-analysis study that reported increased sugar, antioxidants, and several minerals in potato, tomato, and lettuce (Dong et al., 2018). Further, the mitigating action of CO_2_ on Cr VI phytotoxicity at the levels of growth and physiology of rice plants at the vegetative stage had been elucidated (AbdElgawad et al., 2022b). However, there is a lack of information regarding the clear interaction of eCO_2_ and Cr and their synchronous effect on rice yield and grain quality. To this end, as an expected consequence of the effect of eCO_2_ on plants grown in HMs contaminated soil, the current study was designed to fill the gap and investigate the ability of varied CO_2_ levels in mitigating the effect of Cr on grains’ yield and quality of two rice cultivars, Giza 181 and Sakha 106. Results afford a scientific background about rice grain quality under Cr stresses in relation to varying concentrations of CO_2_.

## 2 Materials and methods

### 2.1 Plant growth and treatments

Rice (*Oryza sativa*) seeds (cultivars Sakha 106 and Giza 181) were obtained from Agricultural Research Center, Giza, Egypt. These two cultivars were selected based on their differential responses to heavy metals stress. A homogenous lot of *Oryza sativa* were surface sterilized using sodium hypochlorite (5% v/v; for 20 min). Seeds were grown in a moist perlite. Then seedlings were transplanted into pots (14 cm high and 13 cm in diameter) filled with clay soil. A basal fertilizer was applied at the rate of 1.2 g urea (N content 46%) and 1.2 g of K_2_HPO_4_.3H_2_O. Pots were incubated in a controlled growth chamber (12 h of photoperiod, photosynthetically active radiation of 350 µmol photons m^−2^ s^−1^, 80% of humidity and 28/24°C Day/night temperatures). Rice cultivars were grown in 6 scenarios: 1) ambient CO_2_ (control, 410 ppm CO_2_ (aCO_2_)); 2) aCO_2_ + Cr VI (200 mg/kg soil); 3) aCO_2_ + Cr VI (400 mg/Kg soil); 4) elevated CO_2_ (eCO_2_, 620 ppm); 5) eCO_2_ + Cr VI (200 mg/Kg soil) and 6) eCO_2_ + Cr VI (400 mg/Kg soil). In order to minimize any bias among the cabinets, the experiment had been replicated once again with swapping the two CO_2_ levels among the cabinets. During the entire experiment, CO_2_ was supplied in the airflow of the cabinet and its concentration was continuously monitored with a CO_2_ analyser (WMA-4, PP Systems, Hitchin, UK). A preliminary experiment was conducted with gradient levels of Cr (50-500 mg/Kg soil) to select the most proper concentration of Cr VI. 80% soil water content was kept throughout the experiment. After 3 months dried rice grains were harvested for biochemical analyses. About 100 grains per treatment were milled by a polisher, then unbroken grains and bran were removed using a 1.5 mm sieve. The cleaned milled samples were used for the biochemical analyses. Part of the collected samples were kept in −80°C for enzymes activities investigations. Moreover, soil samples were compiled for chemical analyses.

### 2.2 Cr level analyses

The levels of Cr in rice grains were measured by flow injection hydride generation atomic absorption spectrophotometry (Welsch, 1990). To extract and investigate total Cr, the samples were digested, overnight at 120°C, in HNO_3_ and HCLO_4_. The digestion was stopped when the deep white fumes were released. After that, the levels of Cr were estimated using flow injection hydride generation atomic absorption spectrophotometry (FI-HG-AAS, Perkin Elmer A Analyst 400, CITY, USA) using external calibration (Welsch, 1990). The maximum sensitivity was obtained by using 10% HCl and 0.4% NaBH_4_.

### 2.3 Photosynthetic rate and pigment analysis

The light-saturated photosynthetic rate (*A*sat, μmol CO_2_ m^−2^ s^−1^) was estimated using (LI-COR LI-6400, LI-COR Inc., Lincoln, NE, USA) as outlined previously (AbdElgawad et al., 2015). LI-COR leaf chamber conditions were set according to the climate treatment. Leaf chamber conditions were controlled at 390 ppm CO_2_ and 23.5°C (block temperature) at saturating PAR (1500 μmol m−2 s−1) and ambient relative humidity for the current climate treatment. For elevated CO_2_ concentration, the conditions were controlled at 620 ppm.

### 2.4 Antioxidants analysis

To extract polyphenols and flavonoids, a known weight of fine powdered plant tissues was homogenized in 80% ethanol. The slurry was centrifuged (5000 g for 15 min), and the clear extract was used to quantify total phenolics and flavonoids using Folin-Ciocalteu and AlCl_3_ methods, respectively. Additionally, tocopherols were extracted in hexane, followed by evaporation using CentriVap concentrator (Labconco, Kansas, USA), and the dry pellet was resuspended in hexane. At the end of the extraction, tocopherols were separated, and their levels were determined by HPLC (Shimadzu, ‘s Hertogenbosch, The Netherlands) coupled with a fluorometric detector (excitation at 290 nm and emission at 330 nm). Tocopherols were separated on normal phase conditions, Particil Pac 5 μm column material, length 250 mm, i.d. 4.6 mm. The mobile phase was applied at a flow rate of 0.45 ml min^−1^. Dimethyl tocol (DMT) was used as internal standard (5 ppm). Data were analyzed with Shimadzu Class VP 6.14 software.

In 80% ethanol extract, the total antioxidant capacity (TAC, FRAP) was determined. Centrifugation was done at 14000 g, 4°C, for 25 min, and then the FRAP test [TPTZ (0.01 mM) in HCl (0.04 mM), acetate buffer (0.3 M, pH3.6), and FeCl_3_.6H_2_O (0.02 M)] was performed by using Trolox (0 to 650 M), as already described (Benzie and Strain, 1996).

### 2.5 Sugar metabolism

Sugars levels in rice grain were extracted in 50 mM TAE buffer, pH 7.5, supplemented with a mixture of polyclar (0.15%), Na azide (0.02%), PMSF (2 mM), mercapto-ethanol (1 mM), mannitol (10 mM), and NaHSO_3_ (12 mM). The mixture was centrifuged (15000 g, 4°C, 10 min). Afterwards, a part of the mixture was incubated at 90°C for 5 min, then it was allowed to cool down. Centrifugation was done again (14,000 g, 4°C, 5 min), then the clear supernatant was moved to a mixed bed Dowex column of 300 µL Dowex H^+^, 300 µL Dowex Ac^–^; both 100–200 mesh. Thereafter, elution was done with ddH_2_O, and quantification of different sugars, (glucose, sucrose, raffinose, and fructose) was done by using (HPAEC-PAD) (Verspreet et al., 2012). Sugar separation was done on CarboPac MA1 column. The flow rate of 0.3 mL min−1 of the eluent NaOH gradient (250–700 mM) was applied. The quantification of existing sugars was carried out by comparing the peak areas obtained from calibration curve with those of the corresponding authentic external standards. The internal standard maltotriose which is not naturally present in the samples was used to control the quality of extraction and purification.

For determination of the activities of sugar-related enzymes in rice grains, the samples were extracted in HEPES buffer (100 mM HEPES pH 8.2, 10 mM EDTA, 5 mM MgCl_2_, 15 mM KCl, 2 mM sodium diethyl dithiocarbamate, 5 mM β-mercaptoethanol, 1% PPV) by MagNALyser (Kerr et al., 1985). After centrifugation at 14,000 g and 4°C for 15 min, the clear supernatant was used for activity determination., starch synthase activity was done in a mixture containing glycogen and citrate (Nishi et al., 2001). Meanwhile, amylase activity was detected in a starch solution containing of I_2_/KI (0.05%) in HCl (0.05% as well), and then the reading was taken at 620 nm (Madany and Khalil, 2017).

### 2.6 Protein content

Total proteins were quantified by using the modified semimicro-Kjeldahl methods (Bremner and Hauck, 1982). Albumin, globulin, glutelin and prolamin were assessed using the methods described by Kumamaru et al. (1988). Prolamin contents were determined using Bradford Protein Assay Kit and glutelin content was determined using a bicinchoninic acid Kit.

### 2.7 Organic acids

Organic acids were detected in rice grains by using HPLC. The HPLC system consisted of a liquid chromatographer (Dionex, USA) and a detector (LED, ultimate 3000), in addition to a pump (LPG-3400A), a column thermostat (TCC-3000SD) and an autosampler (EWPS-3000SI). Separation of organic acids was conducted through an Aminex HPH-87 H (300 × 7.8 mm) column coupled with IG Cation H (30 × 4.6) precolumn of Bio-Red firm (at 65°C). Samples were eluted using 0.001 N sulfuric acid (0.6 mL min^-1^) and the detection was done at 210 nm. Data analysis and interpretation were done using chromeleon v.6.8 computer software (Hamad et al., 2015).

### 2.8 Amino acids levels and metabolism

Amino acids were analyzed according to AbdElgawad et al. (2014). Extraction was done by using 100 mg of grain samples in 5 mL of 80% ethanol, and then centrifugation was done (14,000 ×g, 25 min). Afterwards, the supernatant was taken and resuspended in chloroform (5 mL). Detection and quantification of amino acids were done by using UPLC (Waters Acquity, TQD). The aqueous phase was filtered through two Millipore micro filters (0.2 μM pore size). A fixed volume of filtrated supernatant was diluted with the internal standard deuterium labelled l-glutamine- 2,3,3,4,4-d_5_ (C/D/N Isotopes INC, Pointe-Claire, Quebec). Free amino acids were separated on BEH amide column. A gradient mobile phase system consisted of [A: ammonium formate (84%), acetonitrile (10%) acid and formic (6%)], and [B: acetonitrile and formic acid (2%)] at flow rate of 0.3 mL min^−1^.

### 2.9 Fatty acids

Fatty acids levels were determined in grains of treated and non-treated plants by using GC/MS (Hewlett Packard, USA). A know weight of the powdered grains was extracted in chloroform/methanol (2:1, v/v) at 25°C and the lipophilic fractions were centrifuged at 16,000 rpm for 30 min (Hassan et al., 2018). To normalize the extraction efficiency, nonadecanoic acid was used as an internal standard. GC/MS analysis was performed on a Hewlett Packard 6890, MSD 5975 mass spectrometer (Hewlett Packard, Palo Alto, CA, USA), with an HP -5 MS column (30 m × 0.25 mm × 0.25 mm). A 1.0-μL sample was injected using a split mode (split ratio, 1:10). Helium gas was used as a carrier gas at a flow rate of 1.5 mL/min. An electron ionization mode with ionization energy of 70 eV was used for MS detection. The injector and MS transfer line temperatures were set at 220 and 290°C, respectively. The mass scan ranged from 50 to 550 m/z with an Em voltage, 1035 V. The quantification of fatty acids was conducted by comparison of the mass spectrometric ion signal of the target molecule with that of an identical standard. The fatty acid content in the sample is then calculated from the standard curve using analyte/internal standard ion yield ratios (Hassan et al., 2018).

### 2.10 Statistics

The results were expressed as mean ± SD (standard deviation) and analyzed by two-way ANOVA using IBM SPSS Statistical 23 software package (SPSS^®^ Inc., Chicago, IL, USA). Statistical significance of the mean data was assessed by *post-hoc* TukeyHSD (p ≤ 0.05).

## 3 Results

### 3.1 Growth and photosynthesis

**Figure 1:**
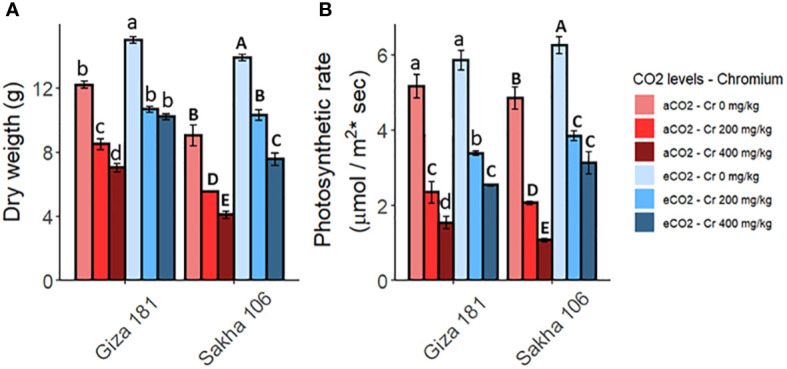
Effect of Chromium (Cr) exposure on dry biomass **(A)** and the rate of photosynthesis **(B)** in rice plants grown under either aCO_2_ (410 ppm) or eCO_2_ (620 ppm). Values are presented as means± standard error of 4 independent replicates. Different letters within the same cultivar represent significant differences between means (Tukey’s Test; P < 0.05).

Soil contamination with Cr greatly inhibited the biomass production and the photosynthetic rate in both rice cultivars, while these reductions were less severe in Giza 181 (**Figure 1**). Under Cr free conditions, eCO_2_ improved the biomass and photosynthetic rate in both cultivars, where Sakha 106 is the most responsive. Further, eCO_2_ effectively recovered the hazardous impact of Cr on growth and photosynthesis of rice plants (**Table S1**). Such mitigating action of eCO_2_ was more obvious in Sakha 106.

### 3.2 Accumulation of Cr^6+^ and grain yield

**Figure 2:**
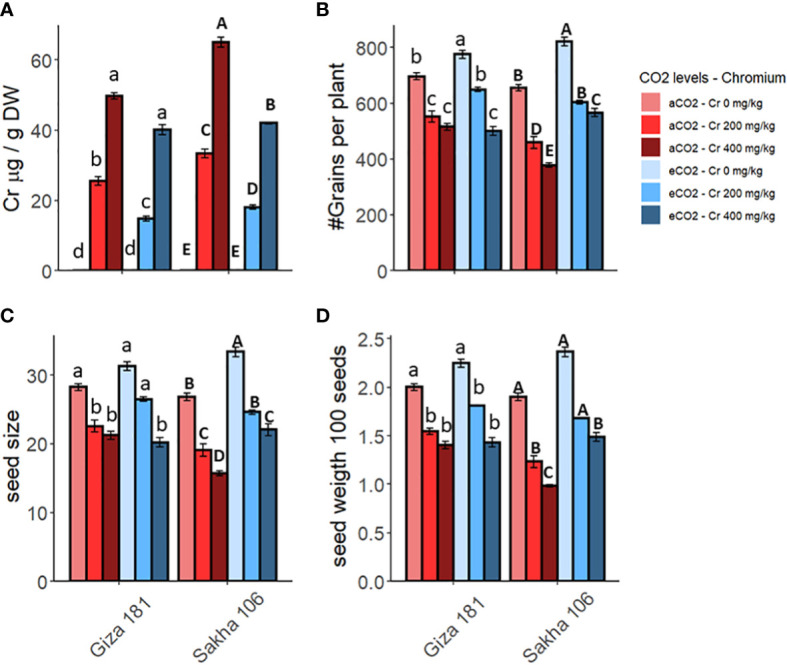
Effect of Chromium (Cr) exposure on Cr accumulation **(A)** and yield parameters **(B–D)** of rice grains produced under either aCO2 (410 ppm) or eCO2 (620 ppm). Values are presented as means ± standard error of 4 independent replicates. Different letters within the same cultivar represent significant differences between means (Tukey's Test; P < 0.05).

The accumulation of Cr^6+^ in the grains of the two rice cultivars is increased by increasing the concentration of Cr in the soil (**Figure 2**). However, Sakha 106 cultivar accumulated higher levels of Cr^6+^ in their grains, compared to Giza 181. The co-application of eCO_2_ with Cr decreased the accumulation of Cr^6+^ in the grains of both cultivars (**Table S1**). For instance, eCO_2_ treatment decreased the levels of Cr^6+^ by about 35 and 20% in grains of Sakha 106 and Giza 181, respectively, at the sever dose of Cr (400 mg Cr^6+^/Kg soil).

Expectedly, soil contamination with Cr significantly inhibited the yield parameters of both rice cultivars, specially under the higher dose of Cr (**Figure 2**). However, yield of Giza 181 cultivar was less affected by Cr accumulation as compared with that of Sakha 106. In this regard, the higher dose of Cr caused 25, 30 and 26% reductions in the numbers of grains per plant, grain size, and grain weight, respectively, in Giza 181 cultivar compared to 41, 48 and 42% in Sakha 106. eCO_2_ caused a positive impact on the measured yield parameters in both cultivars. Further, the co-application of eCO_2_ significantly recovered the negative impact of the lower Cr concentration on both cultivars (**Table S1**) and that for the higher Cr dose in Sakha 106 only.

### 3.3 Elemental composition of rice grains

In general, Cr toxicity considerably retard the elemental composition of rice grains and such effect was more sever at the higher dose of Cr, where Sakha cultivar was the most affected (**Table 1**). For instance, 400 mg Cr^6+^/Kg soil caused significant reductions in the levels of P, S, K, Ca, Zn and Mn in both cultivars and that for Mg, Fe and Cu in Sakha 106 only. In absence of Cr stress, eCO_2_ significantly improved the levels of P in Giza 181 and that for Mg and Ca in both cultivars. eCO_2_ significantly recovered the inhibitory action of Cr stress on the levels of most of the detected elements in both cultivars.

**Table 1:** Effect of Chromium (Cr) exposure on elemental composition of rice grains produced under either aCO_2_ (410 ppm) or eCO_2_ (620 ppm).

Element	aCO_2_			eCO_2_		
	Control	200 mg Cr/Kg soil	400 mg Cr/Kg soil	0 mg Cr/Kg soil	200 mg Cr/Kg soil	400 mg Cr/Kg soil
	Giza 181					
P	42.1 ± 3.7c	43.42 ± 2.2c	83.3 ± 1.39a	64.2 ± 12.8b	63.3 ± 3.8b	64.9 ± 7.0b
S	157.9 ± 20.1b	121.59 ± 6.2c	123.2 ± 3.3c	159.99 ± 16b	177.2 ± 10.4a	168.4 ± 10.6a
K	536 ± 19.3a	504.1 ± 25b	427.8 ± 7.8d	578.2 ± 20.5a	541.2 ± 9.9a	491.7 ± 9c
Mg	126.4 ± 7.3b	101.97 ± 5.2c	122.4 ± 2.8b	151.9 ± 9.1a	151.87 ± 6.0a	145.1 ± 12.4a
Ca	6.9 ± 0.25b	5.83 ± 0.54b	4.13 ± 0.38d	8.66 ± 0.4a	8.59 ± 0.96a	5.17 ± 0.27c
Na	10.94 ± 0.51a	10.41 ± 0.59a	10.97 ± 0.4a	9.56 ± 0.4a	10.06 ± 0.99a	9.94 ± 0.52a
Zn	3.37 ± 0.35a	2.5 ± 0.13b	2.13 ± 0.05b	3.4 ± 0.29a	3.64 ± 0.22a	3.54 ± 0.25a
Fe	1.37 ± 0.08a	1.1 ± 0.05b	1.29 ± 0.03a	1.64 ± 0.09a	1.64 ± 0.07a	1.54 ± 0.13a
Cu	1.76 ± 0.3b	2.04 ± 0.1b	2.3 ± 0.08b	2.04 ± 0.21b	3.41 ± 0.29a	3.01 ± 0.51a
Mn	0.12 ± 0.02c	0.14 ± 0.01a	0.16 ± 0.01b	0.14 ± 0.01b	0.24 ± 0.02a	0.21 ± 0.04a
	Sakha 106					
P	62.9 ± 5.07a	39.08 ± 1.7c	38.67 ± 3.7c	66.59 ± 2.1a	60.86 ± 3.7a	53.8 ± 4.4b
S	166.2 ± 9.0a	122.7 ± 8.5c	96.2 ± 7.2d	174.35 ± 10a	157.06 ± 2.7b	131.3 ± 6.1c
K	582.4 ± 3.5a	421.2 ± 5.8b	379 ± 14.14c	577.6 ± 21a	565.5 ± 12a	431 ± 12.76b
Mg	126.6 ± 6.6b	93.8 ± 3.0c	78.75 ± 5.7d	141.6 ± 3.7a	145.9 ± 2.4a	122.3 ± 14.1b
Ca	6.8 ± 0.94b	4.74 ± 0.19c	4.48 ± 0.26c	9.47 ± 0.87a	7.23 ± 0.42a	6.47 ± 0.68b
Na	10.67 ± 0.5a	10.16 ± 0.58a	10.05 ± 0.9a	9.33 ± 0.4b	9.81 ± 0.97a	9.7 ± 0.51a
Zn	3.47 ± 0.2a	2.12 ± 0.11b	2.04 ± 0.14c	3.65 ± 0.17a	3.3 ± 0.02a	2.81 ± 0.13b
Fe	1.37 ± 0.07a	1.01 ± 0.03b	0.85 ± 0.06c	1.55 ± 0.03a	1.57 ± 0.02a	1.32 ± 0.14a
Cu	2.16 ± 0.12b	1.51 ± 0.1c	3.6 ± 0.19a	1.34 ± 0.2c	1.55 ± 0.2c	3.29 ± 0.32a
Mn	0.15 ± 0.01b	0.11 ± 0.01c	0.25 ± 0.01a	0.09 ± 0.02c	0.11 ± 0.02c	0.23 ± 0.02a

Values are presented as means± standard error of 4 independent replicates. Different letters within the same row indicate significant differences between means (Tukey’s Test; P < 0.05).

### 3.4 Grain protein content

**Figure 3:**
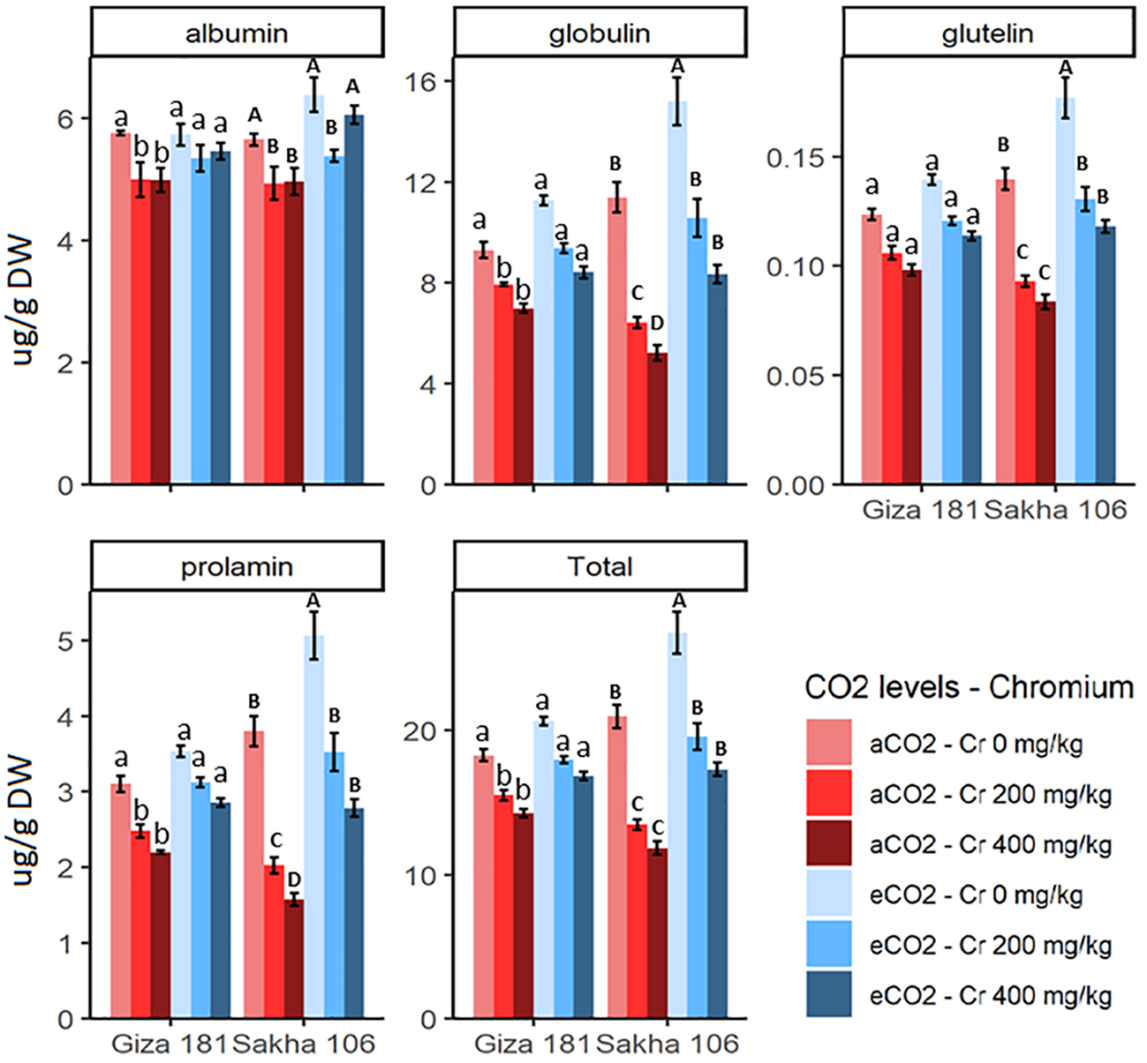
Effect of Chromium (Cr) exposure on protein profile of rice grains produced under either aCO2 (410 ppm) or eCO2 (620 ppm). Values are presented as means± standard error of 4 independent replicates. Different letters within the same cultivar represent significant differences between means (Tukey's Test; P < 0.05).

Cr stress significantly decreased the accumulation of prolamin, globulin, albumin, glutelin and total proteins in grains of Giza 181 and to more extent in Sakha 106 (**Figure 3**). For instance, Cr at concentration of 400 mg/Kg soil decreased the prolamin, globulin and total protein of Sakha 106 and Giza 181 by about 59, 40 and 44%, and 29, 20 and 22%, respectively, as compared with their respective controls. eCO_2_ alone treatment improved the accumulation of globulin, glutelin, prolamin, and total protein in the grains of Sakha 106, but had no significant impact on the protein profile of Giza 181. Under Cr stress, eCO_2_ efficiently recovered the adverse effects of Cr on accumulation of prolamin, globulin and total protein, especially in Sakha 106. In this regard, at sever Cr stress, the levels of grain prolamin and globulin in Sakha 106 and Giza 181 plants grown under eCO_2_ were 1.7 and 1.4-fold, and 1.3 and 1.2-fold, respectively, higher than those found in grains produced under aCO_2_.

### 3.5 Sugars and organic acids

Unlike soluble sugars, Cr stress reduced the starch levels and inhibited the activities of starch synthase in both cultivars (**Table 2**). In unstressed plants, eCO_2_ had no significant impact on the different sugar fractions in Sakha 106 but improved the soluble sugars in Giza 181. However, in Cr-stressed plants, eCO_2_ decreased the Cr-induced accumulation in soluble sugars in Giza 181 and recovered the inhibition in the activities of starch synthase in both cultivars (**Table S1**).

**Table 2:** Effect of Chromium (Cr) exposure on the levels of sugars and organic acids and the activities of sugar metabolizing enzymes in rice grains produced under either aCO_2_ (410 ppm) or eCO_2_ (620 ppm).

Parameter	aCO_2_			eCO_2_		
	Control	200 mg Cr/Kg soil	400 mg Cr/Kg soil	0 mg Cr/Kg soil	200 mg Cr/Kg soil	400 mg Cr/Kg soil
	Giza 181					
Reducing sugars	1.73 ± 0.2c	3.19 ± 0.18a	3.07 ± 0.16a	2.65 ± 0.16b	2.55 ± 0.18b	3.08 ± 0.23a
Non reducing sugars	4.09 ± 0.3c	6.77 ± 0.26a	6.46 ± 0.08a	4.66 ± 0.42b	4.39 ± 0.31b	4.84 ± 0.47b
Total soluble sugars	5.82 ± 0.28c	9.96 ± 0.44a	9.53 ± 0.18a	7.3 ± 0.57b	6.94 ± 0.36b	7.92 ± 0.56b
Starch	751.15 ± 58a	646 ± 39.06a	541.6 ± 33.2c	677.1 ± 29.0a	623.6 ± 28.7b	614.8 ± 3.3b
Amylase	1.8 ± 0.06a	1.45 ± 0.05a	1.54 ± 0.11a	1.11 ± 0.13b	1.81 ± 0.11a	1.72 ± 0.31a
Starch synthase	1.78 ± 0.33b	1.45 ± 0.05c	1.32 ± 0.06c	2.64 ± 0.33a	1.88 ± 0.18b	1.65 ± 0.32b
Succinate	241.7 ± 9.1b	236.7 ± 16.0b	328.06 ± 8.6a	330.2 ± 32.5a	263.4 ± 7.46b	333.7 ± 27.8a
Malate	98.11 ± 1.26a	87.61 ± 2.76a	83.99 ± 4.96a	98.95 ± 3.59a	89.25 ± 4.39a	97.07 ± 8.82a
Citrate	136.8 ± 4.9c	133.0 ± 7c	178.3 ± 4.9a	181 ± 16.7a	148 ± 4.1b	182.1 ± 14a
Lactate	128.7 ± 4.7c	125.6 ± 7.8c	171.2 ± 4.6a	173.5 ± 16a	140.0 ± 3.9b	174 ± 14a
Trans-aconitic	30.64 ± 2.2a	30.32 ± 1.58a	26.91 ± 2.08a	27.24 ± 0.37a	26.07 ± 1.15a	26.39 ± 1.14a
Oxalate	57.06 ± 0.7b	58.27 ± 1.3b	54.28 ± 2.7b	61.9 ± 2.17a	61.28 ± 2.3a	66.48 ± 3.5a
	Sakha 106					
Reducing sugars	2.1 ± 0.2b	4.85 ± 0.25a	3.71 ± 0.05a	2.13 ± 0.11b	3.51 ± 0.17a	3.78 ± 0.41a
Non reducing sugars	4.69 ± 0.81b	6.38 ± 0.33a	6.32 ± 0.6a	5.11 ± 0.35b	6.05 ± 0.52a	5.49 ± 0.54b
Total soluble sugars	6.8 ± 0.73b	11.23 ± 0.57a	10.02 ± 0.58a	7.24 ± 0.26b	9.56 ± 0.37a	9.27 ± 0.94a
Starch	711 ± 46.7a	592.3 ± 48.4b	599.2 ± 18.b	710.4 ± 25.7a	631.7 ± 16b	581.9 ± 45b
Amylase	1.42 ± 0.06a	1.14 ± 0.09c	0.94 ± 0.03c	1.18 ± 0.02c	1.08 ± 0.03c	0.98 ± 0.04c
Starch synthase	1.72 ± 0.03a	0.79 ± 0.03b	0.72 ± 0.03b	1.85 ± 0.32a	1.52 ± 0.14a	1.43 ± 0.27a
Succinate	311.0 ± 17a	298.3 ± 20a	309.8 ± 5.2a	333.4 ± 27.3a	283.6 ± 30.1b	302.5 ± 14a
Malate	91.09 ± 0.28a	81.82 ± 4.07a	82.24 ± 2.02b	89.56 ± 4.58a	81.94 ± 0.35a	85.45 ± 7.25a
Citrate	170 ± 8.03a	157.6 ± 9b	168.5 ± 4.0b	183.4 ± 13a	157.3 ± 15.b	165.1 ± 7.1b
Lactate	163.2 ± 8.5a	150 ± 9.8b	161.7 ± 3.3a	175.0 ± 13a	149.6 ± 15b	158.2 ± 7.a
Trans-aconitic	30.43 ± 1.63a	30.01 ± 3.35a	27.66 ± 2.21b	31.15 ± 0.97a	30.97 ± 0.75a	28.23 ± 1.32a
Oxalate	57.53 ± 3.2a	57.78 ± 5.7a	52.72 ± 0.2a	61.8 ± 0.49a	54.3 ± 0.63b	49.64 ± 1.7b

Values are presented as means± standard error of 4 independent replicates. Different letters within the same row indicate significant differences between means (Tukey’s Test; P < 0.05).

The lower dose of Cr did not exert any significant effect on the concentrations of organic acids in the two cultivars (**Table 2**). The higher dose of Cr, however, improved the accumulation of succinate, citrate and lactate, in Giza 181 and decreased the levels of malate, citrate and trans-aconitate in Sakha 106. eCO_2_ had no significant effect on the levels of organic acids in Sakha 106 plants grown, however in Giza 181 eCO_2_ improved the levels of succinate, citrate, lactate and oxalate. Further, eCO_2_ increased the levels of oxalate in Giza 181 and that for malate and trans-aconitate in Sakha 106 plants grown under 400 mg Cr/Kg soil.

### 3.6 Amino acids composition

Despite the variability in the response to Cr stress among the two cultivars, some amino acids showed distinctive patterns of response (**Table 3**). For instance, lysin and threonine were improved in both cultivars, while valine was sharply decreased. Cultivar specific responses were also recorded in response to eCO_2_ alone treatment. For example, lysine, histidine and aspartate were improved in Sakha 106 but not affected in Giza 181. On contrary, the levels of serine and cystine were decreased in Sakha 106 and unchanged in Giza 181. Under Cr stress, eCO_2_ improved the accumulation of lysine and histidine in both cultivars. Unlike in Giza 181, eCO_2_ decreased the levels of aspartate, valine and tyrosine in Cr-stressed Sakha 106 plants. On the other hand, eCO_2_ decreased the levels of glycine and arginine in Giza 181, but not Sakha 106, under Cr stress.

**Table 3:** Effect of Chromium (Cr) exposure on amino acids profile of rice grains produced under either aCO_2_ (410 ppm) or eCO_2_ (620 ppm).

Amino acids	aCO_2_			eCO_2_		
	Control	200 mg Cr/Kg soil	400 mg Cr/Kg soil	0 mg Cr/Kg soil	200 mg Cr/Kg soil	400 mg Cr/Kg soil
	Giza 181					
Proline	2.45 ± 0.1c	3.3 ± 0.05b	4.5 ± 0.04a	2.03 ± 0.06c	2.66 ± 0.06c	2.4 ± 0.07c
Glycine	0.37 ± 0.01a	0.26 ± 0.01b	0.24 ± 0.02b	0.35 ± 0.03b	0.42 ± 0.02a	0.38 ± 0.04a
Serine	0.27 ± 0.01a	0.29 ± 0.01a	0.17 ± 0.02b	0.32 ± 0a	0.3 ± 0a	0.27 ± 0a
Arginine	0.37 ± 0c	0.45 ± 0.02b	0.26 ± 0.02d	0.47 ± 0.03b	0.51 ± 0.01b	0.46 ± 0b
Ornithine	3.09 ± 0.06b	3.27 ± 0.05b	2.9 ± 0.08b	1.98 ± 0.07c	3.15 ± 0.05b	4.3 ± 0.08a
Glutamine	2.68 ± 0.09b	3.31 ± 0.04a	1.82 ± 0.02c	2.75 ± 0.22b	3.27 ± 0.21a	2.67 ± 0.32b
Glutamate	2.39 ± 0.25b	2.35 ± 0.04b	2.08 ± 0.07b	3.61 ± 0.13a	2.69 ± 0.1b	3.66 ± 0.28a
Aspartate	0.89 ± 0a	0.64 ± 0.01b	0.58 ± 0.01b	0.77 ± 0.04a	0.83 ± 0.02a	0.75 ± 0.02a
Cystine	0.01 ± 0a	0.01 ± 0a	0.01 ± 0a	0.01 ± 0a	0.01 ± 0a	0.01 ± 0a
Asparagine	0.09 ± 0.01a	0.09 ± 0a	0.08 ± 0a	0.09 ± 0a	0.1 ± 0a	0.09 ± 0a
Leucine	0.45 ± 0.06a	0.17 ± 0c	0.15 ± 0.01c	0.29 ± 0.03b	0.17 ± 0c	0.15 ± 0.01c
Lysine	0.29 ± 0a	0.22 ± 0b	0.2 ± 0b	0.25 ± 0.02a	0.28 ± 0.01a	0.25 ± 0.01a
Histidine	0.28 ± 0a	0.21 ± 0b	0.19 ± 0b	0.23 ± 0.02a	0.26 ± 0.01a	0.24 ± 0.01a
Alanine	0.46 ± 0.02d	0.43 ± 0.01d	0.39 ± 0.01c	0.48 ± 0.03c	0.45 ± 0.01d	0.41 ± 0.02d
Isoleucine	0.14 ± 0.02a	0.17 ± 0.01a	0.16 ± 0.02a	0.1 ± 0.01b	0.15 ± 0a	0.14 ± 0.01a
Methionine	0.47 ± 0.01a	0.40 ± 0.01b	0.39 ± 0.02b	0.42 ± 0.06a	0.46 ± 0.01a	0.42 ± 0a
Threonine	0.19 ± 0a	0.14 ± 0b	0.13 ± 0b	0.16 ± 0.01a	0.18 ± 0a	0.16 ± 0a
Valine	0.25 ± 0.01a	0.20 ± 0.0b	0.19 ± 0.0b	0.26 ± 0.02a	0.24 ± 0a	0.22 ± 0.01a
Phenylalanine	0.28 ± 0.03a	0.32 ± 0.0a	0.29 ± 0.02a	0.25 ± 0b	0.3 ± 0.01a	0.27 ± 0.01a
Tyrosine	0.67 ± 0.03a	0.67 ± 0.02a	0.61 ± 0.04a	0.55 ± 0.05a	0.68 ± 0.01a	0.62 ± 0.01a
	Sakha 106					
Proline	2.68 ± 0.16b	2.68 ± 0.05b	3.58 ± 0.2a	2.36 ± 0.17b	2.68 ± 0.08c	3.67 ± 0.21a
Glycine	0.48 ± 0.03a	0.35 ± 0.01b	0.31 ± 0.02c	0.26 ± 0.01c	0.35 ± 0.01b	0.31 ± 0.01c
Serine	0.31 ± 0b	0.3 ± 0.01a	0.13 ± 0.01b	0.23 ± 0.01a	0.28 ± 0a	0.25 ± 0.01a
Arginine	1.75 ± 0.02a	2.34 ± 0.06a	1.09 ± 0.15b	2.29 ± 0.08a	2.31 ± 0.02a	2.07 ± 0.12a
Ornithine	3.66 ± 0.29c	3.66 ± 0.18c	4.74 ± 0.5a	4.11 ± 0.19b	4.12 ± 0.15b	5.24 ± 0.42a
Glutamine	4.98 ± 0.08a	5.94 ± 0.14a	4.31 ± 0.12b	5.6 ± 0.28a	5.03 ± 0.12a	4.53 ± 0.17b
Glutamate	2.25 ± 0.15b	2.03 ± 0.07b	1.45 ± 0.1c	2.34 ± 0.08b	1.87 ± 0.02c	1.69 ± 0.02c
Aspartate	0.31 ± 0.01c	0.58 ± 0.08a	0.56 ± 0.17a	0.37 ± 0.03b	0.48 ± 0.06b	0.45 ± 0.12b
Cystine	0.15 ± 0.03a	0.02 ± 0d	0.02 ± 0.01d	0.04 ± 0.02c	0.09 ± 0.01b	0.08 ± 0.03b
Asparagine	0.02 ± 0c	0.02 ± 0c	0.02 ± 0c	0.15 ± 0a	0.1 ± 0b	0.09 ± 0b
Leucine	0.21 ± 0.01c	0.15 ± 0.01d	0.13 ± 0.01d	0.12 ± 0.05d	0.26 ± 0.06b	0.3 ± 0.05b
Lysine	0.02 ± 0e	0.05 ± 0d	0.05 ± 0.01d	0.13 ± 0.01c	0.26 ± 0.01a	0.24 ± 0.01a
Histidine	0.02 ± 0c	0.03 ± 0c	0.03 ± 0c	0.12 ± 0.01b	0.23 ± 0.01a	0.21 ± 0.02a
Alanine	0.65 ± 0.1c	0.13 ± 0.01e	0.13 ± 0.03e	1.36 ± 0.36b	2.28 ± 0.19a	1.91 ± 0.7a
Isoleucine	0.12 ± 0.01a	0.13 ± 0a	0.12 ± 0.01a	0.13 ± 0a	0.12 ± 0.01a	0.12 ± 0.01a
Methionine	0.19 ± 0.01b	0.35 ± 0.03a	0.33 ± 0.07a	0.2 ± 0.01b	0.28 ± 0.04a	0.26 ± 0.07a
Threonine	0.08 ± 0b	0.18 ± 0.01a	0.16 ± 0.02a	0.09 ± 0.01b	0.2 ± 0.01a	0.18 ± 0.02a
Valine	1.23 ± 0.18a	0.26 ± 0.04c	0.26 ± 0.11c	0.5 ± 0.12b	0.12 ± 0.04d	0.13 ± 0.08d
Phenylalanine	1.09 ± 0.15a	0.39 ± 0.02c	0.36 ± 0.03c	0.56 ± 0.05b	0.47 ± 0.03b	0.43 ± 0.06b
Tyrosine	0.36 ± 0.01c	0.53 ± 0.04a	0.5 ± 0.08a	0.37 ± 0.01c	0.44 ± 0.05b	0.41 ± 0.09b

Values are presented as means± standard error of 4 independent replicates. Different letters within the same row indicate significant differences between means (Tukey’s Test; P < 0.05).

### 3.7 Fatty acids composition

Cr stress, at both levels, significantly decreased the accumulation of the majority of the detected saturated and unsaturated fatty acids in both rice cultivars (**Table 4**). However, the negative impact of Cr stress on the total unsaturated acids was more evident on Giza 181, specially at the higher dose of Cr. On contrary, eCO_2_ alone treatment exerted a positive impact on the accumulation of most of the individual fatty acids in Sakha 106 and to less extent in Giza 181. Interestingly, Cr stressed plants grown under eCO_2_ accumulated higher levels of the unsaturated fatty acids C20:2 and C24:1, as compared to their respective Cr alone treatments. Further, eCO_2_ improved the accumulation of C16:1, C16:2, C16:3, C18:1 and C18:3 in Cr-stressed Sakha 106.

**Table 4:** Effect of Chromium (Cr) exposure on fatty acids profile of rice grains produced under either aCO_2_ (410 ppm) or eCO_2_ (620 ppm).

Fatty acids	aCO_2_			eCO_2_		
	Control	200 mg Cr/Kg soil	400 mg Cr/Kg soil	0 mg Cr/Kg soil	200 mg Cr/Kg soil	400 mg Cr/Kg soil
	Giza 181					
Dodecanoic (C12:0)	275.8 ± 19.7a	272.9 ± 14.2a	242.2 ± 18.7b	245.1 ± 3.2b	234.6 ± 10b	237.4 ± 10.2b
Tetradecanoic (C14:0)	237 ± 14.6b	251.4 ± 9.5b	246.3 ± 12b	312.7 ± 18a	316.8 ± 11a	360 ± 27.6a
Pentadecanoic (C15:0)	340.3 ± 0.4a	301.1 ± 24a	271 ± 20.6b	343.1 ± 7.4a	318 ± 26.9a	358.8 ± 38.7a
Hexadecanoic (C16:0)	4473 ± 80a	4050 ± 144a	3953 ± 193b	4628 ± 167a	4704 ± 104a	4249 ± 183a
Hexadecanoic (C16:1	229.9 ± 8b	216 ± 57b	200 ± 19b	274 ± 27a	184.4 ± 20c	217 ± 10b
Hexadecadienoic (C16:2)	93.8 ± 3.4b	88.1 ± 23.4b	81.7 ± 7.83b	112.2 ± 11a	75.2 ± 8.5c	88.64 ± 4.4b
Hexadecatrienoic (C16:3)	80.9 ± 3.0b	76.06 ± 20b	70.4 ± 6.7b	96.8 ± 9.5a	64.9 ± 7.3c	76.45 ± 3.8b
Heptadecanoic (C17:0)	151.2 ± 6.4a	136.4 ± 7.1b	146.1 ± 7.1a	149.5 ± 12.a	117.3 ± 5.1c	131 ± 12.2b
Octadecanoic (C18:0)	381 ± 14.8a	352.5 ± 57b	346.3 ± 13b	424 ± 20a	301.7 ± 25.b	348 ± 19b
Octadecenoic (18:1)	2610 ± 257a	2066 ± 290a	1895 ± 107b	2683 ± 189a	1946 ± 95b	2136 ± 99a
Octadecatrienoic (C18:3)	3466 ± 284a	2860 ± 426b	2669 ± 140.1b	3644 ± 238a	2625.34 ± 154b	2922 ± 142b
Eicosanoic (C20:0)	164.2 ± 4.84	157.3 ± 3.36c	142.26 ± 3.5c	236.7 ± 46.5b	288.0 ± 46.9a	250.6 ± 81.5b
Eicosadienoic (C20:2)	167.11 ± 12.8a	137.16 ± 2.14b	124.26 ± 0.9b	138.1 ± 19.34b	167.4 ± 8.76a	163.3 ± 26.38a
Docosanoic (C22:0)	112.92 ± 1.1b	88.8 ± 1.25c	113.9 ± 11.1b	157.6 ± 5.48a	105.0 ± 15.6b	125.59 ± 3.9b
Tetracosanoic (C24:0)	162.6 ± 16.5a	154.16 ± 12a	137.1 ± 20.0b	186.1 ± 16.3a	139.8 ± 7.3b	125.3 ± 11.6c
Tetracosenoic (C24:1)	64.05 ± 9.6b	63.34 ± 4.0b	55.07 ± 9.2b	84.2 ± 3.73a	81.68 ± 7a	75.7 ± 12.8a
Pentacosanoic (C25:0)	58.85 ± 0.18c	64.42 ± 9.19b	63.23 ± 1.98b	89.84 ± 8.83a	77.02 ± 3.75a	54.34 ± 3.28c
Hexacosanoic (26:0)	53.6 ± 9.5c	76.6 ± 10b	71.3 ± 8.8b	95.4 ± 14.9a	72.3 ± 4.7b	44.0 ± 4.4c
Total Saturated FA	6411.6 ± 103a	5906.5 ± 84c	5734 ± 211c	6869 ± 120a	6675 ± 118a	6287 ± 273b
Total Unsaturated FA	6631 ± 549a	5431 ± 792b	5025 ± 283c	6937 ± 468a	5080 ± 277c	5603 ± 263b
	Sakha 106					
Dodecanoic (C12:0)	273.9 ± 14.6a	270.0 ± 30a	248 ± 19.9b	280.7 ± 8.7a	278.7 ± 6.7a	254 ± 11.9b
Tetradecanoic (C14:0)	243.8 ± 14a	249.9 ± 24a	225.5 ± 17a	276.4 ± 13a	209.9 ± 3.2b	192.6 ± 5.2b
Pentadecanoic (C15:0)	317.2 ± 13a	275 ± 22b	271 ± 20.3b	296 ± 31b	259 ± 1.7C	288 ± 26b
Hexadecanoic (C16:0)	4268 ± 297a	3740 ± 242b	3775. ± 505b	4553 ± 174a	4257 ± 200a	3820 ± 278b
Hexadecanoic (C16:1	208.8 ± 14c	160.9 ± 8.3d	161.3 ± 7.7d	316.3 ± 3.6a	259.3 ± 31b	196.6 ± 29c
Hexadecadienoic (C16:2)	85.2 ± 6.06b	65.68 ± 3.42c	65.87 ± 3.1c	129.1 ± 1.4a	105.8 ± 12.7a	80.2 ± 11.8b
Hexadecatrienoic (C16:3)	73.5 ± 5.2b	56.6 ± 2.9b	56.8 ± 2.7b	111.3 ± 1.2a	91.29 ± 11a	89.2 ± 5.5b
Heptadecanoic (C17:0)	136.9 ± 7.3a	135.0 ± 15b	140.2 ± 24a	140.1 ± 4.3a	139.3 ± 3.3a	141 ± 15.4a
Octadecanoic (C18:0)	345.7 ± 17b	295 ± 7.34b	301 ± 31b	456 ± 7.7a	398 ± 33.0a	337 ± 41.8b
Octadecenoic (18:1)	1817 ± 148.1b	1787.79 ± 57.42c	1701.26 ± 41b	2261 ± 14a	2407.3 ± 246.3a	2052 ± 151a
Octadecatrienoic (C18:3)	2594 ± 189b	2445 ± 48.6b	2133.2 ± 93.8c	3304.1 ± 18.5a	3310.5 ± 316a	2808.1 ± 56.5b
Eicosanoic (C20:0)	179.39 ± 9.4c	159.99 ± 2.46c	144.59 ± 2.07c	246.9 ± 32.6b	311.9 ± 25.8a	269.9 ± 54.22a
Eicosadienoic (C20:2)	173.1 ± 24.6a	138.7 ± 1.99b	112.1 ± 1.41c	196.31 ± 9.89a	178.7 ± 2.71a	143.6 ± 10.24b
Docosanoic (C22:0)	122.3 ± 5.2b	116.7 ± 10.8b	115.11 ± 12.3b	170.64 ± 4.6a	169.11 ± 5.6a	162.9 ± 9.66a
Tetracosanoic (C24:0)	142.6 ± 6.67b	121.6 ± 1.5c	100.9 ± 4.99c	152.7 ± 18.2a	120.9 ± 9.8c	107.7 ± 16.4c
Tetracosenoic (C24:1)	69.36 ± 6.7a	65.8 ± 1.2b	58.6 ± 3.75b	88.0 ± 0.23a	73.5 ± 1.39a	67.1 ± 1.89b
Pentacosanoic (C25:0)	81.45 ± 6.54a	77.39 ± 2.94a	68.61 ± 1.81b	90.53 ± 6.34a	69.81 ± 0.19b	62.46 ± 3.55b
Hexacosanoic (26:0)	93.5 ± 11.8a	88.9 ± 6.63a	78.5 ± 0.31b	92.9 ± 12.4a	66.11 ± 1.1c	57.81 ± 8.0d
Total Saturated FA	6205 ± 325b	5531 ± 226c	5471 ± 467d	6757 ± 149a	6280 ± 214b	5695 ± 299c
Total Unsaturated FA	4948 ± 329c	4664 ± 120d	4232 ± 143e	6295 ± 38a	6335 ± 598a	5348 ± 175b

Values are presented as means± standard error of 4 independent replicates. Different letters within the same row indicate significant differences between means (Tukey’s Test; P < 0.05).

### 3.8 Molecular antioxidants and total antioxidant capacity

**Figure 4:**
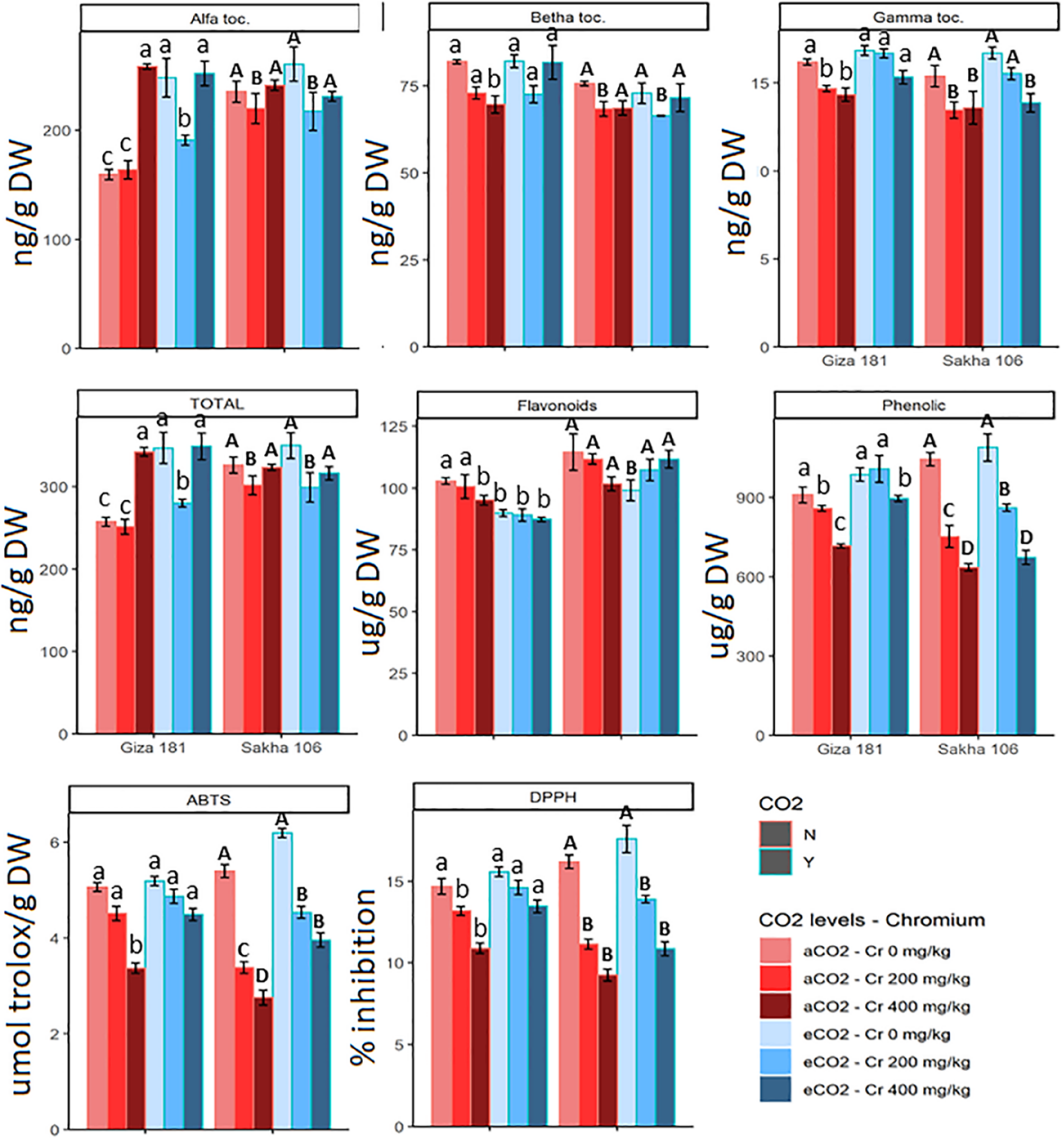
Effect of Chromium (Cr) exposure on the levels of molecular antioxidants and total antioxidant capacities of rice grains produced under either aCO2 (410 ppm) or eCO2 (620 ppm). Values are presented as means± standard error of 4 independent replicates. Different letters within the same cultivar represent significant differences between means (Tukey's Test; P < 0.05).

The impact of Cr toxicity on accumulation of total phenolics and TAC was more sever at the higher Cr concentration and was more evident for Sakha 106 cultivar (**Figure 4**). For instance, Sakha 106 plants grown in soil contaminated with 400 mg Cr^6+^/Kg soil showed about 39, 43 and 49% reductions in the content of total phenolics and the FRAP and ABTS radical scavenging activities, respectively, as compared with 21, 26 and 34% inhibitions in case of Giza 181. The sever Cr dose improved the total tocopherols in Giza 181 but not in Sakha 106, however it reduced the gamma tocopherol in both cultivars. eCO_2_ alone treatment improved the total tocopherols in Giza 181 but had no significant effects on the levels of flavonoids and total phenolics or the FRAP and ABTS radical scavenging activities in both cultivars. However, under Cr stress, eCO_2_ mitigated the negative impacts of Cr on phenolics accumulation and TAC in both cultivars.

## 4 Discussion

Rice represents the major source for carbohydrates in the diet of about one half of the world population. Unfortunately, the production of rice is threatened by the presence of contaminants in water and soils. In this regard, Cr has been reported as a major pollutant that affect the yield and quality of rice grains in affected areas (Ma et al., 2016; Basit et al., 2021). eCO_2_ has been reported to ameliorate the negative impacts of some heavy metals, e.g., Hg, Cu, Cd and Pb, on several plant species (Guo et al., 2011; Kim and Kang, 2011; Saleh et al., 2021). In a recent study, AbdElgawad et al. (2022b) had addressed the mitigating action of CO_2_ on Cr VI phytotoxicity at the levels of growth and physiology of rice plants at the vegetative stage, however the implication on the quality of rice grains had not been studied. Herein, we have evaluated the mitigating action of eCO_2_ on the yield and quality of two rice cultivars, Giza 181 and Sakha 106, grown under chromate (Cr VI), the most stable and bioavailable form of Cr in the environment (Vitale et al., 1997; Oliveira, 2012).

**Figure 5:**
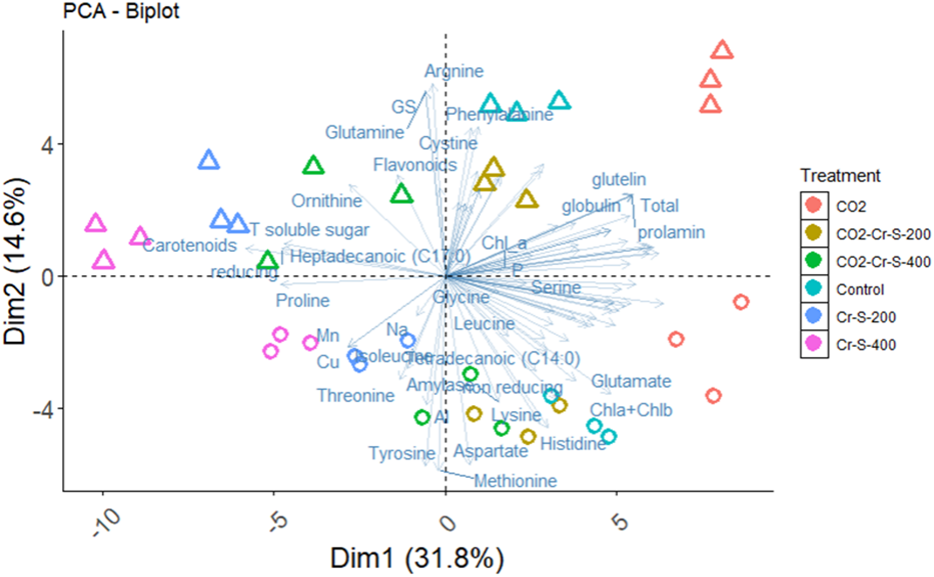
Principal component analysis (PCA) of biochemical parameters of rice grains produced under Chromium (Cr) and either aCO2 (410 ppm) or eCO2 (620 ppm). Variances explained by the first two components (PC1 and PC2) appear in parentheses.

Cr is inactively absorbed by the plant roots and negatively affect the uptake of essential mineral nutrients, accordingly, this will reduce the plant growth (Wakeel et al., 2020). The present data showed that Cr stress significantly inhibited the whole plant biomass of both rice cultivars at the harvest time, in a concentration dependent manner. This reduction could be ascribed to the sharp inhibition noted in the photosynthetic rate of rice plants as affected by Cr (**Figure 1**). Similarly, Cr application in soil (100 to 500 mg kg^−1^) markedly reduced the growth, photosynthetic pigments, and photosynthetic rate of rice plants (Hussain et al., 2018; AbdElgawad et al., 2022b). Further, Basit et al. (2021) reported that Cr adversely affected the biomass production and photosynthetic rate and induced the accumulation of ROS and cell damage. Notably, eCO_2_ mitigated the negative impact of Cr on dry matter. eCO_2_ has been reported to improve the growth of HMs stressed plants by enhancing the photosynthetic C assimilation, as a substrate for Rubisco, and by inhibiting photorespiration, thereby, reducing the production of H_2_O_2_ and the provoked cell damage (Saleh et al., 2019; AbdElgawad et al., 2022a). In accordance, the present results indicated that eCO_2_ significantly ameliorated the hazardous impact of Cr on the photosynthetic rate of both cultivars with Sakha 106 is the more responsive to eCO_2_. Such impact was confirmed by PCA which reveals a separation on the bases of Cr treatment on the PC1, which explains 31.8% of the variance, meanwhile Cr combined with eCO_2_ treatments seems closer to the control plants (**Figure 5**).

Similar to the present results, the accumulation of Cr in grains and the reduction in yield of rice plants as affected by soil pollution with Cr has been reported (Sundaramoorthy et al., 2010; Zhou et al., 2019). In consistence with its ameliorating action against the Cr-induced reduction in growth and photosynthesis, eCO_2_ caused a notable increase in the yield parameters of both rice cultivars grown under Cr and reduced the accumulation of Cr in the produced grains. Similarly, eCO_2_ has been recorded to enhance the yield and ear number in rice grown in Hg polluted soil (Mao et al., 2021). Despite the absence of literature regarding the impact of eCO_2_ on the accumulation of Cr in rice grains, Guo et al. (2011) reported that grains of rice and wheat plants grown in soils polluted with Cd or Cu accumulated higher Cd and less Cu compared to those produced under aCO_2_. On the other hand, the ability of eCO_2_ to reduce the accumulation of Cr in vegetative tissues of rice has been recently reported (AbdElgawad et al., 2022b). Further, AbdElgawad et al. (2022a) has pointed to the ability of eCO_2_, alone or combined with arbuscular mycorrhizal fungi, to reduce the accumulation of As^III^ in wheat and soybean plants. They have ascribed the reduced uptake of these HMs under eCO_2_ to the lower stomatal conductance and the higher exudation of polyphenols and organic acid into the soil, relative to the control. This explanation was supported by the fact that polyphenols and organic acids can form complexes with HMs, thereby, reduced their bioavailability (Schwab et al., 2005; Campbell and Nordstrom, 2014).

The nutritional quality of rice grains is related to their carbohydrate, protein and lipid compositions (Yibo et al., 2022). In addition, secondary metabolites, e.g., polyphenols, and minerals, e.g., Fe and Zn, that exist in the rice grain are beneficial to human health (Zhao et al., 2020). To assess the impact of Cr and/or eCO_2_ on the nutritional value of rice grains we have performed a metabolic profiling of carbohydrates, proteins, amino acids, fatty acids, minerals and antioxidant metabolites. The results indicated a negative impact for Cr stress, especially at the highest dose, on the grains’ nutritional value in Giza 181 and to more extent in Sakha 106 in term of the following: 1) increased accumulation of Cr; 2) reduced starch content; 3) decreased levels of individual and total proteins and several amino acids; 4) reduced levels of several unsaturated fatty acids; 5) lower levels of mineral nutrients such as Fe and Zn; 6) reduced content of polyphenols and TAC. Interestingly, growing plants under eCO_2_ efficiently antagonized the adverse effects of Cr on the nutritional value of rice grains. In this regard, Sharma et al. (1995) reported that Cr VI (0.05-1.00 mM) greatly reduced the grain yield of *T. aestivum* plants, where at the highest dose, the plants failed to form seeds. Maize plants treated with Cr VI at concentrations of 30-150 μmol L^-1^ showed increased accumulation of Cr in grains and decreased yield attributes, in a dose dependent manner (Anjum et al., 2017). Further, Singh et al. (2020) have reported that growing two varieties of chickpea in soil treated with Cr VI (60 μM and 120 μM) significantly reduced the yield attributes (e.g. pod number, grain yield per plant) and grain proteins.

In fact, most carbon that integrate to the nutritional components of the rice grain is imported as photoassimilates from the photosynthetic leaves, particularly the flag leaf, during the grain-filling, (Yoshida, 1981). Further, the correlation between C assimilation and yield in rice is likely (Sharkey et al., 2000). Thus, the negative impact of Cr on the yield and nutritional value of rice grains could be a logical consequence of its adverse action on plant biochemical attributes. In this regard, Cr has been reported to induce oxidative damage and to inhibit many physiological and biochemical aspects in plants, including photosynthesis and C and N metabolism (Sangwan et al., 2014; Shahid et al., 2017; Hussain et al., 2018). Further, by its negative impact on starch synthesizing enzymes, e.g., starch synthase, Cr could retard the sink strength of the developed grains, thus affecting its filling with C and N metabolites (Mohapatra et al., 2009). On the other hand, the ameliorating impact of eCO_2_ against Cr-induced reduction in yield and nutritional compositions could be attributed to the enhancement of photosynthetic C assimilation, which will improve the biosynthesis of photoassimilates and hence retrieve the source strength. In this regard, Mao et al. (2021) reported that eCO_2_ significantly promoted the light saturated CO_2_ assimilation rate in rice plants, during both flowering and grain filling periods, which improved the yield attributes. Högy et al. (2009) revealed that eCO_2_ caused enhancements in the levels of non-structural carbohydrates and lipids but decreased the protein content in wheat grains. Elevated CO_2_ improved grain size and weight but did not significantly affect its nitrogen and amylose content in wild and domesticated rice genotypes (Rahman et al., 2021). Besides eCO_2_ has found to enhance the activity of starch synthesizing enzymes and improve the accumulation of starch in wheat and maize plants grown under mercuric oxide nanoparticles (AbdElgawad et al., 2020). Thus, it could be hypothesized that the positive impact of eCO_2_ on the nutritional value of grains is more evident under stressful conditions that retard the source strength of the photosynthetic leaves and/or the sink strength of the developing grains.

## 5 Conclusion

Based on the results of the present study it could be concluded that Cr stress significantly inhibited the growth and photosynthetic and grain yield and quality of rice plants. However, growing plants under of eCO_2_ could mitigate the negative effects of Cr at the levels of growth, physiology, and yield production. Cr exposure reduces the quality of rice grains by inducing the accumulation of Cr and reducing the levels of nutritionally important metabolites such as starch, proteins, unsaturated fatty acids, antioxidant molecules, and minerals. Interestingly, eCO_2_ reduced the accumulation of Cr in rice grains and improved the grain quality under Cr toxicity. To this end, growing rice under CO_2_-enriched environment could reduce the toxicity hazards of Cr and support the production and quality of the produced grains.

## Data availability statement

The raw data supporting the conclusions of this article will be made available by the authors, without undue reservation.

## Author contributions

Conception and design of study: HA, JvD, GB, AS. Acquisition of data: HA, JvD, AS. Analysis and/or interpretation of data: AM, MA, JvD, AS. Drafting the manuscript: HA, AM, AS. Revising the manuscript critically for important intellectual content: HA, JvD, AS, GB. All authors contributed to the article and approved the submitted version.

## References

[B1] AbdElgawad H. De Vos D. Zinta G. Domagalska M. A. Beemster G. T. S. Asard H. (2015). Grassland species differentially regulate proline concentrations under future climate conditions: An integrated biochemical and modelling approach. New Phytol. 208, 354–369. doi: 10.1111/nph.13481 26037253 PMC4744684

[B2] AbdElgawad H. El-Sawah A. M. Mohammed A. E. Alotaibi M. O. Yehia R. S. Selim S.. (2022a). Increasing atmospheric CO_2_ differentially supports arsenite stress mitigating impact of arbuscular mycorrhizal fungi in wheat and soybean plants. Chemosphere 296, 134044. doi: 10.1016/j.chemosphere.2022.134044 35202662

[B3] Abdelgawad H. Farfan-vignolo E. R. Vos D. Asard H. (2015). Elevated CO_2_ mitigates drought and temperature-induced oxidative stress differently in grasses and legumes. Plant Sci. 231, 1–10. doi: 10.1016/j.plantsci.2014.11.001 25575986

[B4] AbdElgawad H. Hassan Y. M. Alotaibi M. O. Mohammed A. E. Saleh A. M. (2020). C3 and C4 plant systems respond differently to the concurrent challenges of mercuric oxide nanoparticles and future climate CO_2_ . Sci. Total Environ. 749, 142356. doi: 10.1016/j.scitotenv.2020.142356 33370918

[B5] AbdElgawad H. Peshev D. Zinta G. den Ende W. Janssens I. A. Asard H. (2014). Climate extreme effects on the chemical composition of temperate grassland species under ambient and elevated CO2: A comparison of fructan and non-fructan accumulators. PloS One 9, e92044. doi: 10.1371/journal.pone.0092044 24670435 PMC3966776

[B6] AbdElgawad H. Sheteiwy M. S. Saleh A. M. Mohammed A. E. Alotaibi M. O. Beemster G. T. S.. (2022b). Elevated CO2 differentially mitigates chromium (VI) toxicity in two rice cultivars by modulating mineral homeostasis and improving redox status. Chemosphere 307, 135880. doi: 10.1016/j.chemosphere.2022.135880 35964713

[B7] Anjum S. A. Ashraf U. Imran K. Tanveer M. Shahid M. Shakoor A.. (2017). Phyto-toxicity of chromium in maize: Oxidative damage, osmolyte accumulation, anti-oxidative defense and chromium uptake. Pedosphere 27, 262–273. doi: 10.1016/S1002-0160(17)60315-1

[B8] Arif N. Sharma N. C. Yadav V. Ramawat N. Dubey N. K. Tripathi D. K.. (2019). Understanding heavy metal stress in a rice crop: Toxicity, tolerance mechanisms, and amelioration strategies. J. Plant Biol. 62, 239–253. doi: 10.1007/s12374-019-0112-4

[B9] Basit F. Chen M. Ahmed T. Shahid M. Noman M. Liu J.. (2021). Seed priming with brassinosteroids alleviates chromium stress in rice cultivars *via* improving ROS metabolism and antioxidant defense response at biochemical and molecular levels. Antioxidants 10, 1089. doi: 10.3390/antiox10071089 34356322 PMC8301181

[B10] Becker C. Kläring H.-P. (2016). CO_2_ enrichment can produce high red leaf lettuce yield while increasing most flavonoid glycoside and some caffeic acid derivative concentrations. Food Chem. 199, 736–745. doi: 10.1016/j.foodchem.2015.12.059 26776031

[B11] Benzie I. F. F. Strain J. J. (1996). The ferric reducing ability of plasma (FRAP) as a measure of “antioxidant power”: The FRAP assay. Anal. Biochem. 239, 70–76. doi: 10.1006/abio.1996.0292 8660627

[B12] Bremner J. M. Hauck R. D. (1982). Advances in methodology for research on nitrogen transformations in soils. Nitrogen Agric. soils 22, 467–502. doi: 10.2134/agronmonogr22.c13

[B13] Campbell K. M. Nordstrom D. K. (2014). Arsenic speciation and sorption in natural environments. Rev. Mineral Geochem. 79, 185–216. doi: 10.2138/rmg.2014.79.3

[B14] Castro R. O. Trujillo M. M. Bucio J. L. Cervantes C. Dubrovsky J. (2007). Effects of dichromate on growth and root system architecture of *Arabidopsis thaliana* seedlings. Plant Sci. 172, 684–691. doi: 10.1016/j.plantsci.2006.11.004

[B15] Dong J. Gruda N. Lam S. K. Li X. Duan Z. (2018). Effects of elevated CO_2_ on nutritional quality of vegetables: A review. Front. Plant Sci. 9. doi: 10.3389/fpls.2018.00924 PMC6104417 30158939

[B16] Eleftheriou E. P. Adamakis I. D. S. Panteris E. Fatsiou M. (2015). Chromium-induced ultrastructural changes and oxidative stress in roots of *Arabidopsis thaliana* . Int. J. Mol. Sci. 16, 15852–15871. doi: 10.3390/ijms160715852 26184178 PMC4519928

[B17] Giri S. Singh A. K. (2017). Human health risk assessment due to dietary intake of heavy metals through rice in the mining areas of singhbhum copper belt, India. Environ. Sci. pollut. Res. 24, 14945–14956. doi: 10.1007/s11356-017-9039-9 28484981

[B18] Guo H. Zhu J. Zhou H. Sun Y. Yin Y. Pei D.. (2011). Elevated CO_2_ levels affects the concentrations of copper and cadmium in crops grown in soil contaminated with heavy metals under fully open-air field conditions. Environ. Sci. Technol. 45, 6997–7003. doi: 10.1021/es2001584 21770376

[B19] Hamad I. AbdElgawad H. Al Jaouni S. Zinta G. Asard H. Hassan S.. (2015). Metabolic analysis of various date palm fruit (*Phoenix dactylifera* l.) cultivars from Saudi Arabia to assess their nutritional quality. Molecules 20, 13620–13641. doi: 10.3390/molecules200813620 26225946 PMC6331958

[B20] Hassan M. O. Saleh A. M. AbdElgawad H. (2018). *Sonchus oleraceus* residue improves nutritive and health-promoting value of common bean ( *Phaseolus vulgaris* l.): A metabolic study. J. Agric. Food Chem. 66, 2092–2100. doi: 10.1021/acs.jafc.7b05821 29455523

[B21] Högy P. Wieser H. Köhler P. Schwadorf K. Breuer J. Franzaring J.. (2009). Effects of elevated CO2 on grain yield and quality of wheat: Results from a 3-year free-air CO_2_ enrichment experiment. Plant Biol. 11, 60–69. doi: 10.1111/j.1438-8677.2009.00230.x 19778369

[B22] Hussain A. Ali S. Rizwan M. Zia ur Rehman M. Hameed A. Hafeez F.. (2018). Role of zinc–lysine on growth and chromium uptake in rice plants under cr stress. J. Plant Growth Regul. 37, 1413–1422. doi: 10.1007/s00344-018-9831-x

[B23] Kerr P. S. Rufty T. W. Huber S. C. (1985). Endogenous rhythms in photosynthesis, sucrose phosphate synthase activity, and stomatal resistance in leaves of soybean (*Glycine max* [L.] merr.). Plant Physiol. 77, 275–280. doi: 10.1104/pp.77.2.275 16664041 PMC1064502

[B24] Kim S. Kang H. (2011). Effects of elevated CO_2_ and Pb on phytoextraction and enzyme activity. Water. Air. Soil pollut. 219, 365–375. doi: 10.1007/s11270-010-0713-5

[B25] Kumamaru T. Satoh H. Iwata N. Omura T. Ogawa M. Tanaka K. (1988). Mutants for rice storage proteins. Theor. Appl. Genet. 76, 11–16. doi: 10.1007/BF00288825 24231976

[B26] Luo X. S. Zhang D. Hu Z. Liu C. Zhao Z. Sun W.. (2019). Effects of elevated carbon dioxide on metal transport in soil-crop system: Results from a field rice and wheat experiment. J. Soils Sediments 19, 3742–3748. doi: 10.1007/s11368-019-02329-z

[B27] Madany M. M. Y. Khalil R. R. (2017). Seed priming with ascorbic acid or calcium chloride mitigates the adverse effects of drought stress in sunflower (*Helianthus annuus* l.) seedlings. Egypt J. Exp. Biol. 13, 119–133. doi: 10.5455/egyj

[B28] Ma J. Lv C. Xu M. Chen G. Lv C. Gao Z. (2016). Photosynthesis performance, antioxidant enzymes, and ultrastructural analyses of rice seedlings under chromium stress. Environ. Sci. pollut. Res. 23, 1768–1778. doi: 10.1007/s11356-015-5439-x 26396015

[B29] Mao Q. Tang L. Ji W. Rennenberg H. Hu B. Ma M. (2021). Elevated CO_2_ and soil mercury stress affect photosynthetic characteristics and mercury accumulation of rice. Ecotoxicol. Environ. Saf. 208, 111605. doi: 10.1016/j.ecoenv.2020.111605 33396125

[B30] Martínez-Trujillo M. Méndez-Bravo A. Ortiz-Castro R. Hernández-Madrigal F. Ibarra-Laclette E. Ruiz-Herrera L. F.. (2014). Chromate alters root system architecture and activates expression of genes involved in iron homeostasis and signaling in *Arabidopsis thaliana* . Plant Mol. Biol. 86, 35–50. doi: 10.1007/s11103-014-0210-0 24928490

[B31] Mohapatra P. K. Sarkar R. K. Kuanar S. R. (2009). Starch synthesizing enzymes and sink strength of grains of contrasting rice cultivars. Plant Sci. 176, 256–263. doi: 10.1016/j.plantsci.2008.11.001

[B32] Nishi A. Nakamura Y. Tanaka N. Satoh H. (2001). Biochemical and genetic analysis of the effects of amylose-extender mutation in rice endosperm. Plant Physiol. 127, 459–472. doi: 10.1104/pp.010127.BEII 11598221 PMC125082

[B33] Oliveira H. (2012). Chromium as an environmental pollutant: Insights on induced plant toxicity. J. Bot. 2012, 1–8. doi: 10.1155/2012/375843

[B34] Panhwar Q. A. Naher U. A. Radziah O. Shamshuddin J. Razi I. M. Dipti S. S.. (2015). Quality and antioxidant activity of rice grown on alluvial soil amended with zn, Cu and Mo. South Afr. J. Bot. 98, 77–83. doi: 10.1016/j.sajb.2015.01.021

[B35] Pérez-López U. Robredo A. Lacuesta M. Sgherri C. Muñoz-Rueda A. Navari-Izzo F.. (2009). The oxidative stress caused by salinity in two barley cultivars is mitigated by elevated CO_2_ . Physiol. Plant 135, 29–42. doi: 10.1111/j.1399-3054.2008.01174.x 19121097

[B36] Rahman S. Copeland L. Atwell B. J. Roberts T. H. (2021). Elevated CO2 differentially affects the properties of grain from wild and domesticated rice. J. Cereal Sci. 100, 103227. doi: 10.1016/j.jcs.2021.103227

[B37] Rai P. K. Lee S. S. Zhang M. Tsang Y. F. Kim K. H. (2019). Heavy metals in food crops: Health risks, fate, mechanisms, and management. Environ. Int. 125, 365–385. doi: 10.1016/j.envint.2019.01.067 30743144

[B38] Rosado-Porto D. Ratering S. Cardinale M. Maisinger C. Moser G. Deppe M.. (2021). Elevated atmospheric CO2 modifies mostly the metabolic active rhizosphere soil microbiome in the giessen FACE experiment. Microb. Ecol 83(3), 619–634. doi: 10.1007/s00248-021-01791-y PMC8979872 34148108

[B39] Saleh A. M. Hassan Y. M. Habeeb T. H. Alkhalaf A. A. Hozzein W. N. Selim S.. (2021). Interactive effects of mercuric oxide nanoparticles and future climate CO_2_ on maize plant. J. Hazard Mater. 401. doi: 10.1016/j.jhazmat.2020.123849 33113748

[B40] Saleh A. M. Hassan Y. M. Selim S. AbdElgawad H. (2019). NiO-nanoparticles induce reduced phytotoxic hazards in wheat (*Triticum aestivum* l.) grown under future climate CO_2_ . Chemosphere 220, 1047–1057. doi: 10.1016/J.CHEMOSPHERE.2019.01.023 33395791

[B41] Sangwan P. Kumar V. Joshi U. N. (2014). Effect of chromium (VI) toxicity on enzymes of nitrogen metabolism in clusterbean (*Cyamopsis tetragonoloba* l.). Enzyme Res. 2014. doi: 10.1155/2014/784036 PMC3976926 24744916

[B42] Schwab A. P. He Y. Banks M. K. (2005). The influence of organic ligands on the retention of lead in soil. Chemosphere 61, 856–866. doi: 10.1016/j.chemosphere.2005.04.098 15979688

[B43] Senthil-Nathan S. (2021). Effects of elevated CO_2_ on resistant and susceptible rice cultivar and its primary host, brown planthopper (BPH), nilaparvata lugens (Stål). Sci. Rep. 11, 8905. doi: 10.1038/s41598-021-87992-4 33903626 PMC8076292

[B44] Shabbaj I. I. Abdelgawad H. Balkhyour M. A. Tammar A. Madany M. M. Y. (2022). Elevated CO_2_ differentially mitigated oxidative stress induced by indium oxide nanoparticles in young and old leaves of C3 and C4 crops. Antioxidants 11. doi: 10.3390/antiox11020308 PMC8868301 35204191

[B45] Shahid M. Shamshad S. Rafiq M. Khalid S. Bibi I. Niazi N. K.. (2017). Chromium speciation, bioavailability, uptake, toxicity and detoxification in soil-plant system: A review. Chemosphere 178, 513–533. doi: 10.1016/j.chemosphere.2017.03.074 28347915

[B46] Sharkey T. D. Laporte M. M. Kruger E. L. (2000). “Will increased photosynthetic efficiency lead to increased yield in rice?,” in Studies in plant science 7, 73–86. (Elsevier)

[B47] Sharma D. C. Chatterjee C. Sharma C. P. (1995). Chromium accumulation and its effects on wheat (*Triticum aestivum* l. cv. HD 2204) metabolism. Plant Sci. 111, 145–151. doi: 10.1016/0168-9452(95)04230-R

[B48] Sharma A. Kapoor D. Wang J. Shahzad B. Kumar V. Bali A. S.. (2020). Chromium bioaccumulation and its impacts on plants: An overview. Plants 9. doi: 10.3390/plants9010100 PMC7020214 31941115

[B49] Singh D. Sharma N. L. Singh C. K. Kumar Sarkar S. Singh I. Lal Dotaniya M. (2020). Effect of chromium (VI) toxicity on morpho-physiological characteristics, yield, and yield components of two chickpea (*Cicer arietinum* l.) varieties. PloS One 15. doi: 10.1371/journal.pone.0243032 PMC7714171 33270694

[B50] Srivastava D. Tiwari M. Dutta P. Singh P. Chawda K. Kumari M.. (2021). Chromium stress in plants: Toxicity, tolerance and phytoremediation. Sustain 13. doi: 10.3390/su13094629

[B51] Sundaramoorthy P. Chidambaram A. Ganesh K. S. Unnikannan P. Baskaran L. (2010). Chromium stress in paddy:(i) nutrient status of paddy under chromium stress;(ii) phytoremediation of chromium by aquatic and terrestrial weeds. C. R. Biol. 333, 597–607. doi: 10.1016/j.crvi.2010.03.002 20688280

[B52] Tchounwou P. B. Yedjou C. G. Patlolla A. K. Sutton D. J. (2012). Heavy metal toxicity and the environment. EXS 101, 133–164. doi: 10.1007/978-3-7643-8340-4_6 22945569 PMC4144270

[B53] Verspreet J. Pollet A. Cuyvers S. Vergauwen R. den Ende W. Delcour J. A.. (2012). A simple and accurate method for determining wheat grain fructan content and average degree of polymerization. J. Agric. Food Chem. 60, 2102–2107. doi: 10.1021/jf204774n 22324634

[B54] Vitale R. J. Mussoline G. R. Rinehimer K. A. (1997). Environmental monitoring of chromium in air, soil, and water. Regul. Toxicol. Pharmacol. 26, S80–S85. doi: 10.1006/rtph.1997.1144 9380841

[B55] Wakeel A. Xu M. Gan Y. (2020). Chromium-induced reactive oxygen species accumulation by altering the enzymatic antioxidant system and associated cytotoxic, genotoxic, ultrastructural, and photosynthetic changes in plants. Int. J. Mol. Sci. 21. doi: 10.3390/ijms21030728 PMC7037945 31979101

[B56] Wang J. Liu X. Zhang X. Li L. Lam S. K. Pan G. (2019). Changes in plant c, n and p ratios under elevated [CO_2_] and canopy warming in a rice-winter wheat rotation system. Sci. Rep. 9. doi: 10.1038/s41598-019-41944-1 PMC6443658 30931987

[B57] Welsch F. P. (1990). Trace elements determination of arsenic and selenium using continuous-flow hydride generation atomic absorption spectrophotometry (HG-AAS). Qual. Assur. Man. branch geochem., 38–45.

[B58] Yibo C. Zhidong W. Chongrong W. Hong L. Daoqiang H. Degui Z.. (2022). Comparisons of metabolic profiles for carbohydrates, amino acids, lipids, fragrance and flavones during grain development in indica rice cultivars. Rice Sci. 29, 155–165. doi: 10.1016/j.rsci.2022.01.004

[B59] Yoshida S. (1981). Fundamentals of rice crop science. Int. Rice Res. Inst.

[B60] Zhao M. Lin Y. Chen H. (2020). Improving nutritional quality of rice for human health. Theor. Appl. Genet. 133, 1397–1413. doi: 10.1007/s00122-019-03530-x 31915876

[B61] Zhou J. Chen H. Tao Y. Thring R. W. Mao J. (2019). Biochar amendment of chromium-polluted paddy soil suppresses greenhouse gas emissions and decreases chromium uptake by rice grain. J. Soils Sediments 19, 1756–1766. doi: 10.1007/s11368-018-2170-5

[B62] Zhu X. Lin L. Shao J. Yang Y. Jiang X. Zhang Q. (2008). “Effects of zinc and chromium stresses on quality of rice grain,” in 2nd International Conference on Bioinformatics and Biomedical Engineering, iCBBE 2008. 4475–4479 (Shanghai: IEEE Computer Society). doi: 10.1109/ICBBE.2008.612

