# Supplementary material for: The impact of chromium toxicity on the yield and quality of rice grains produced under ambient and elevated levels of CO2
Source: Front Plant Sci. 2023 Mar 7;14:1019859. doi: 10.3389/fpls.2023.1019859 (PMC10027917; doi:10.3389/fpls.2023.1019859)
Supplement: Supplementary file 1 [file Table_1.docx]

**Table S1:** A two-way ANOVA for the effect of Cr, eCO_2_ treatments and their interaction on the different measured parameters in rice grains (numbers represent F values; ns = non-significant; *=P < 0.05; **P < 0.01; ***=P < 0.001).

| **Parameter** | **Giza 181** | | |  | **Sakha 106** | | |
| --- | --- | --- | --- | --- | --- | --- | --- |
|  | **Cr** | **eCO_2_** | **Cr x eCO_2_** |  | **Cr** | **eCO_2_** | **Cr x eCO_2_** |
| Growth/yield |  |  |  |  |  |  |  |
| Cr ug g DW | 433.679^***^ | 29.889^***^ | 7.531^**^ |  | 702.66^***^ | 120.47^***^ | 33.73^***^ |
| Seed weight | 65.69^***^ | 12.37^**^ | 4.26^*^ |  | 92.987^***^ | 71.541^**^ | 5.087^*^ |
| DW Plant | 80.283^***^ | 63.625^***^ | 7.065^**^ |  | 41.579^***^ | 72.824^***^ | 7.541^**^ |
| Grai^ns^ per plant | 42.5^***^ | 7.177^*^ | 3.941^ns^ |  | 67.218^***^ | 71.884^*^ | 4.031^*^ |
| Seed size | 35.901^***^ | 5.079^*^ | 3.935^ns^ |  | 53.289^***^ | 44.68^*^ | 5.108^*^ |
| Protein |  |  |  |  |  |  |  |
| Glutelin | 19.503^***^ | 20.228^***^ | 0.012^ns^ |  | 22.165^***^ | 23.751^***^ | 0.017^ns^ |
| Globulin | 25.078^***^ | 28.722^***^ | 0.339^ns^ |  | 21.548^***^ | 19.336^***^ | 0.129^ns^ |
| Albumin | 1.873^ns^ | 0.974^ns^ | 0.307^ns^ |  | 3.095^*^ | 7.088^*^ | 0.432^ns^ |
| Photosynthesis related paramters |  |  |  |  |  |  |  |
| Photosynthesis | 51.53^***^ | 1.986^ns^ | 4.276^*^ |  | 51.904^***^ | 36.958^***^ | 4.7905^*^ |
| Chl b | 14.77^***^ | 27.11^***^ | 0.54^ns^ |  | 88.047^***^ | 2.802^ns^ | 9.232^**^ |
| Chl a | 2.527^ns^ | 0.75^ns^ | 0.848^ns^ |  | 13.82^***^ | 29.02^***^ | 13.96^***^ |
| Chla + Chlb | 59.221^***^ | 0.288^ns^ | 3.633^ns^ |  | 22.081^***^ | 35.493^***^ | 7.886^**^ |
| Caretenoids | 56.831^***^ | 28.375^***^ | 1.953^ns^ |  | 23.938^***^ | 9.614^***^ | 2.649^ns^ |
| Antioxidants |  |  |  |  |  |  |  |
| DPPH | 10.594^**^ | 9.605^**^ | 0.892^ns^ |  | 36.086^***^ | 8.367^**^ | 0.411^ns^ |
| ABTS | 16.472^***^ | 9.682^**^ | 3.023^ns^ |  | 62.515^***^ | 31.992^***^ | 0.471^ns^ |
| Flavenoids | 0.739^ns^ | 9.28^*^ | 0.184^ns^ |  | 0.089^ns^ | 0.281^ns^ | 1.408^ns^ |
| Alpha toc | 11.102^***^ | 7.071^*^ | 4.131^*^ |  | 0.989^ns^ | 0.057^ns^ | 0.396^ns^ |
| Beta toc | 2.091^ns^ | 1.1^ns^ | 1.153^ns^ |  | 1.421^ns^ | 0.034^ns^ | 0.287^ns^ |
| Gamma Toc | 5.687^**^ | 9.009^*^ | 0.99^ns^ |  | 2.83^ns^ | 2.259^*^ | 0.412^ns^ |
| Mineral |  |  |  |  |  |  |  |
| P | 6.934^**^ | 2.232^ns^ | 6.211^*^ |  | 14.039^***^ | 20.162^***^ | 3.082^*^ |
| S | 0.586^ns^ | 11.269^**^ | 2.581^ns^ |  | 26.241^***^ | 16.463^***^ | 1.934^ns^ |
| K | 17.534^***^ | 12.192^**^ | 0.369^ns^ |  | 86.66^***^ | 34.67^***^ | 16.15^***^ |
| Mg | 1.244# | 26.32^***^ | 1.845^ns^ |  | 11.135^***^ | 40.075^***^ | 3.721^ns^ |
| Ca | 20.23^***^ | 18.89^**^ | 1.37^ns^ |  | 10.048^**^ | 21.46^***^ | 0.153^ns^ |
| Na | 0.083^ns^ | 3.473^ns^ | 0.366^ns^ |  | 0.02^ns^ | 1.519^ns^ | 0.358^ns^ |
| Zn | 2.658^ns^ | 19.583^***^ | 4.678^*^ |  | 35.113^***^ | 37.843^***^ | 6.417^**^ |
| Fe | 1.449^ns^ | 30.008^***^ | 2.041^ns^ |  | 13.056^***^ | 44.796^***^ | 3.658^ns^ |
| Cu | 5.071^*^ | 11.12^**^ | 1.805^ns^ |  | 42.411^***^ | 3.783^*^ | 1.822^ns^ |
| Mn | 5.071^*^ | 11.12^**^ | 1.805^ns^ |  | 42.411^***^ | 3.783^*^ | 1.822^ns^ |
| Sugars |  |  |  |  |  |  |  |
| Reducing sugars | 12.015^***^ | 0.425^ns^ | 8.508^**^ |  | 45.712^***^ | 4.993^ns^ | 6.291^**^ |
| Non reducing sugars | 6.588^**^ | 49.64^***^ | 6.597^*^ |  | 0.457^ns^ | 4.18^***^ | 4.472^*^ |
| Total soluble sugars | 15.588^***^ | 9.268^**^ | 14.933^***^ | | 16.432^***^ | 1.705^**^ | 1.48^ns^ |
| Starch | 7.185^**^ | 0.074^ns^ | 2.154^ns^ |  | 6.277^**^ | 0.057^ns^ | 0.323^ns^ |
| Amylase | 0.843^ns^ | 0.154^ns^ | 6.448^**^ |  | 24.454^***^ | 4.468^*^ | 4.494^*^ |
| Starch synthase | 4.699^*^ | 7.221^*^ | 4.011^*^ |  | 9.205^**^ | 12.602^**^ | 3.991^ns^ |
| Succinate | 8.51^**^ | 6.37^*^ | 2.435^ns^ |  | 1.512^ns^ | 0.042^ns^ | 0.309^ns^ |
| Malate | 2.372^ns^ | 1.685^ns^ | 0.978^ns^ |  | 2.481^ns^ | 0.034^ns^ | 0.185^ns^ |
| Citrate | 7.742^**^ | 6.824^*^ | 2.248^ns^ |  | 1.79^ns^ | 0.122^ns^ | 0.327^ns^ |
| Lactate | 8.119^**^ | 6.601^*^ | 2.34^ns^ |  | 1.648^ns^ | 0.077^ns^ | 0.318^ns^ |
| Transaconitic acid | 1.137^ns^ | 4.639^*^ | 0.792^ns^ |  | 1.137^ns^ | 4.639^ns^ | 0.005^ns^ |
| Oxalate | 0.073^ns^ | 12.687^**^ | 2.199^ns^ |  | 4.728^*^ | 0.107^ns^ | 1.256^ns^ |
| Amino acids |  |  |  |  |  |  |  |
| Proline | 175.6^***^ | 392.42^***^ | 97.33^***^ |  | 28.818^***^ | 0.391^ns^ | 0.923^ns^ |
| Glycine | 2.369^ns^ | 21.936^***^ | 7.661^**^ |  | 7.312^**^ | 34.524^***^ | 35.45^ns^ |
| Serine | 35.95^***^ | 41.31^***^ | 10.34^**^ |  | 64.664^***^ | 1.741^ns^ | 61.53^ns^ |
| Arginine | 22.843^***^ | 73.18^***^ | 8.168^**^ |  | 35.88^***^ | 46.68^***^ | 16.54^***^ |
| Ornithine | 133.484^***^ | 1.177^ns^ | 183.091^***^ | | 8.085^***^ | 3.243^ns^ | 0.003^ns^ |
| Glutamine | 15.727^***^ | 3.812^ns^ | 3.439^*^ |  | 23.487^***^ | 0.032^ns^ | 11.6^**^ |
| Glutamate | 4.192^*^ | 55.39^***^ | 6.835^**^ |  | 35.103^***^ | 0.602^ns^ | 2.705^ns^ |
| Aspartate | 33.86^***^ | 20.46^***^ | 34.19^***^ |  | 2.342^ns^ | 0.457^ns^ | 0.455^ns^ |
| Cystine | 7.623^**^ | 1.772^ns^ | 3.506^ns^ |  | 3.567^ns^ | 0.186^ns^ | 13.59^***^ |
| Asparagine | 1.067^ns^ | 2.467^ns^ | 0.923^ns^ |  | 99^***^ | 264^***^ | 60.58^***^ |
| Leucine | 38.848^***^ | 5.743^*^ | 5.743^*^ |  | 1.472^ns^ | 2.427^ns^ | 5.523^**^ |
| Lysine | 10.112^**^ | 5.772^*^ | 16.374^***^ | | 59.95^***^ | 763.92^***^ | 23.05^***^ |
| Histidine | 10.581^**^ | 5.402^*^ | 19.198^***^ | | 16.64^***^ | 343.58^***^ | 12.05^***^ |
| Alanine | 6.337^**^ | 3.183^ns^ | 0^ns^ |  | 0.226^ns^ | 32.15^***^ | 2.493^ns^ |
| Isoleucine | 6.545^**^ | 6.192^**^ | 0.126^ns^ |  | 0.702^ns^ | 0.034^ns^ | 0.377^ns^ |
| Methionine | 1.74^ns^ | 0.081^ns^ | 1.489^ns^ |  | 3.735^ns^ | 1.203^ns^ | 0.451^ns^ |
| Threonine | 10.358^**^ | 5.598^*^ | 17.802^***^ | | 38.261^***^ | 2.389^ns^ | 0.294^ns^ |
| Valine | 11.887^***^ | 7.87^*^ | 0.189^ns^ |  | 25.73^***^ | 14.099^**^ | 5.327^*^ |
| Phenylalanine | 3.715^ns^ | 2.706^ns^ | 0.106^ns^ |  | 22.648^***^ | 4.712^ns^ | 12.24^**^ |
| Tyrosine | 2.977^ns^ | 1.842^ns^ | 2.825^ns^ |  | 2.354^ns^ | 1.384^ns^ | 0.607^ns^ |
| GS | 15.727^***^ | 3.812^***^ | 3.439^ns^ |  | 23.487^***^ | 0.032^ns^ | 11.6^***^ |
| GDH | 99.35^***^ | 73.86^***^ | 20.98^***^ |  | 1.533^ns^ | 0.465^ns^ | 4.81^*^ |
| Fatty acids |  |  |  |  |  |  |  |
| Dodecanoic (C12:0) | 1.137^ns^ | 4.639^*^ | 0.792^ns^ |  | 1.332^ns^ | 0.23^ns^ | 0.005^ns^ |
| Tetradecanoic (C14:0) | 1.495^ns^ | 38.613^***^ | 1.206^ns^ |  | 5.848^**^ | 1.201^ns^ | 3.555^ns^ |
| Pentadecanoic (C15:0) | 1.061^ns^ | 3.44^ns^ | 1.841^ns^ |  | 1.798^ns^ | 0.139^ns^ | 0.444^ns^ |
| Hexadecanoic (C16:0) | 4.481^*^ | 8.886^**^ | 1.44^ns^ |  | 2.128^ns^ | 1.299^ns^ | 0.302^ns^ |
| Hexadecanoic (C16:1 | 1.879^ns^ | 0.184^ns^ | 0.9^ns^ |  | 9.786^**^ | 26.587^***^ | 2.126^ns^ |
| Hexadecadienoic (C16:2) | 1.879^ns^ | 0.184^ns^ | 0.9^ns^ |  | 9.786^**^ | 26.587^***^ | 2.126^ns^ |
| Hexadecatrienoic (C16:3) | 1.879^ns^ | 0.184^ns^ | 0.9^ns^ |  | 9.786^**^ | 26.587^***^ | 2.126^ns^ |
| Heptadecanoic (C17:0) | 3.581^ns^ | 2.726^ns^ | 0.527^ns^ |  | 0.032^ns^ | 0.062^ns^ | 0.008^ns^ |
| Octadecanoic (C18:0) | 3.603^ns^ | 0.005^ns^ | 1.305^ns^ |  | 4.864^*^ | 14.728^**^ | 1.188^ns^ |
| Octadecenoic (18:1) | 7.454^**^ | 0.174^ns^ | 0.453^ns^ |  | 1.419^ns^ | 18.062^**^ | 0.503^ns^ |
| Octadecatrienoic (C18:3) | 6.464^**^ | 0.101^ns^ | 0.539^ns^ |  | 5.299^*^ | 33.529^***^ | 0.204^ns^ |
| Eicosanoic (C20:0) | 0.217^ns^ | 8.777^**^ | 0.234^ns^ |  | 0.579^ns^ | 24.942^***^ | 1.172^ns^ |
| Eicosadienoic (C20:2) | 0.228^ns^ | 1.244^ns^ | 3.122^ns^ |  | 11.778^**^ | 10.846^***^ | 0.259^ns^ |
| Docosanoic (C22:0) | 11.204^***^ | 13.224^**^ | 2.418^ns^ |  | 0.37^ns^ | 48.95^***^ | 0.042^ns^ |
| Tetracosanoic (C24:0) | 4.472^*^ | 0.006^ns^ | 1.053^ns^ |  | 7.425^**^ | 0.339^ns^ | 0.12^ns^ |
| Tetracosenoic (C24:1) | 0.617^ns^ | 8.324^*^ | 0.011^ns^ |  | 11.26^***^ | 18.018^**^ | 1.704^ns^ |
| Pentacosanoic (C25:0) | 4.158^*^ | 6.297^*^ | 6.247^**^ |  | 11.863^***^ | 0.202^ns^ | 2.384^ns^ |
| Hexacosanoic (26:0) | 2.092^ns^ | 0.193^ns^ | 6.89^**^ |  | 4.756^*^ | 4.8^*^ | 1.119^ns^ |
| Total saturated FA | 6.548^**^ | 0.207^ns^ | 0.459^ns^ |  | 4.659^*^ | 4.361^*^ | 0.395^ns^ |
| Total unsaturated FA | 6.548^**^ | 0.207^ns^ | 0.498^ns^ |  | 4.535^*^ | 32.004^***^ | 0.437^ns^ |
